# Selective synthesis of rebaudioside M2 through structure-guided engineering of glycosyltransferase UGT94D1

**DOI:** 10.3389/fbioe.2024.1334427

**Published:** 2024-02-05

**Authors:** Lifeng Yang, Mengliang Yang, Zhiwei Deng, Xiaodong Hou, Xiangting Zheng, Qian Ping, Yijian Rao, Jinsong Shi, Yan Zhang

**Affiliations:** ^1^ School of Life Sciences and Health Engineering, Jiangnan University, Wuxi, China; ^2^ School of Chemical and Material Engineering, Jiangnan University, Wuxi, China; ^3^ School of Biotechnology, Jiangnan University, Wuxi, China; ^4^ Key Laboratory of Carbohydrate Chemistry and Biotechnology, Ministry of Education, School of Life Sciences and Health Engineering, Jiangnan University, Wuxi, China

**Keywords:** steviol glycoside, rebaudioside M2, glycosyltransferase, UGT94D1, protein engineering

## Abstract

Rebaudioside M2 (Reb M2), a novel steviol glycoside derivative, has limited industrial applications due to its low synthetic yield and selectivity. Herein, we identify UGT94D1 as a selective glycosyltransferase for rebaudioside D (Reb D), leading to the production of a mono *β*-1,6-glycosylated derivative, Reb M2. A variant UGT94D1-F119I/D188P was developed through protein engineering. This mutant exhibited a 6.33-fold improvement in catalytic efficiency, and produced Reb M2 with 92% yield. Moreover, molecular dynamics simulations demonstrated that UGT94D1-F119I/D188P exhibited a shorter distance between the nucleophilic oxygen (OH6) of the substrate Reb D and uridine diphosphate glucose, along with an increased O^phosphate^-C1-O^acceptor^ angle, thus improving the catalytic activity of the enzyme. Therefore, this study provides an efficient method for the selective synthesis of Reb M2 and paves the way for its applications in various fields.

## 1 Introduction

Steviol glycosides (SGs), which are derived from the plant *Stevia rebaudiana*, have become a popular alternative to sucrose and artificial sweeteners due to their low-calorie content and high sweetness (Ahmed et al., 2011; DuBois and Prakash, 2012; Gantait et al., 2015; Perrier et al., 2018). Importantly, toxicological studies conducted on SGs have found no evidence of teratogenic, carcinogenic or mutagenic effects, supporting their safe consumption (Ceunen and Geuns, 2013; Libik-Konieczny et al., 2021). To date, researchers have identified over 60 SGs, including steviol, rebaudioside A (Reb A), rebaudioside D (Reb D) and rebaudioside M (Reb M). Each of these SGs has a characteristic level of sweetness and taste as well as specific biological activities due to variations in the positions and quantities of sugars attached to their C-19 and/or C-13 positions (Bhardwaj et al., 2020; Ceunen and Geuns, 2013; Gerwig et al., 2016; Ohtani et al., 1992; te Poele et al., 2018; Tian et al., 2022). Among these known SGs, rebaudioside M2 (Reb M2) (Prakash, et al., 2014a), an isomer of Reb M, has a *β* 1→6 glycosidic bond at its C-19 position, which leads to distinct characteristics and a high economic value (Hellfritsch et al., 2012; Prakash, et al., 2014b; Olsson et al., 2016; Purkayastha et al., 2016). Reb M2 was first identified as a byproduct of the bioconversion of Reb A by the glycosyltransferase UGTSL2 (Prakash, et al., 2014a). The yield of Reb M2 from this process is limited, and subsequent studies have aimed to improve the yield by utilizing UGTSL2 and a sucrose synthase (*St*SUS1). These efforts resulted in yields in excess of 10 g/L (Li et al., 2016), but the amount of Reb M2 produced was still not sufficient to meet the demands of the food industry. Therefore, there is a need for the development of more efficient and selective methods for the production of Reb M2.

Glycosyltransferases (GTs) are enzymes that catalyze the glycosylation of compounds in plant organisms (Lombard et al., 2014; Nidetzky et al., 2018). They transfer the activated sugar donor onto the sugar acceptor, resulting in the formation of glycosylated products. Uridine diphosphate glycosyltransferases (UGTs) (Yonekura-Sakakibara and Hanada, 2011; Zhang et al., 2020), which rely on uridine diphosphate sugar donors, belong to the GT1 multigene family (Paquette et al., 2003), comprising approximately half of the entire GTs family. UGTs have demonstrated exceptional stereo- and regio-selectivity in glycosylation of various natural products (Caputi et al., 2012; Wen et al., 2018; Li et al., 2020; Teze et al., 2021), showcasing their significant potential in the preparation of steviol glycoside derivatives. In our previous research (Ping et al., 2022), the glycosylation of Reb A to synthesize Reb D2 through the construction of a *β* 1→6 glycosidic bond at the C-19 position of Reb A was facilitated by a UGT known as UGT94D1, which was originally identified from *Sesamum indicum* (Noguchi et al., 2008; Ono et al., 2020; Brandt et al., 2021). Hence, it is plausible to achieve a highly selective and efficient synthesis of Reb M2 through the forming of a similar *β* 1→6 glycosidic bond using Reb D as the substrate.

In this study, we demonstrate that Reb M2 can be selectively synthesized by glycosyltransferase UGT94D1 from Reb D via the construction of a *β* 1→6 glycosidic bond at the C-19 position. Its conversion efficiency was greatly improved by the creation of the variant UGT94D1-F119I/D188P through protein engineering, which resulted in a 6.33-fold increase. Efficient production of Reb M2 was realized by coupling this mutant with sucrose synthase (Schmölzer et al., 2016) *At*SuSy in a cascade reaction system to realize the regeneration the glycosyl donor uridine diphosphate glucose (UDPG), resulting in a yield of 92% and generating 23.08 mM (29.79 mg/mL) of Reb M2. Therefore, in this study, we not only developed an efficient method for the selective synthesis of Reb M2 but also paved the way for its future applications.

## 2 Materials and methods

### 2.1 Construction of plasmids and bacterial strains

A list of the bacterial strains and plasmids utilized in this study can be found in Supplementary Table S1. The open reading frames encoding glycosyltransferase UGT94D1 (accession number: XP_011076907.1) from *S. indicum* and sucrose synthase *At*SuSy (accession number: NP_001031915) from *Arabidopsis thaliana* were synthesized and codon-optimized for expression in *E. coli* by Exsyn-bio (Wuxi, China). *Escherichia coli* Top10 was utilized for plasmid construction, while *E. coli* BL21 (DE3) strain was used for protein expression. The expression vectors used were the plasmids pET-21b (+) and pACYCDuet-1. Mutations in UGT94D1 were accomplished through polymerase chain reaction, with the relevant primers listed in Supplementary Table S2. Sequencing was conducted to confirm all insertions and mutations in this process. Macklin Biotechnology (China) supplied Reb D, and other chemicals were obtained from Energy Chemical (Shanghai, China) or China National Pharmaceutical Group Corporation (Shanghai, China).

### 2.2 Protein expression and purification

Recombinant protein expression was performed essentially as described previously (Ping et al., 2022). *Escherichia coli* BL21 (DE3) with the relevant plasmids were cultured in 2 × YT medium with 100 mg/L ampicillin, incubated at 37°C with shaking. Protein expression was induced by adding 0.1 mM isopropyl-*β*-D-thiogalactopyranoside when the optical density at 600 nm reached 0.6 to 0.8, followed by incubation at 18°C for 8 h. After centrifugation, the cells were collected and suspended in a buffer solution containing 250 mM NaCl, 5% glycerol, 10 mM imidazole, and 50 mM Tris-HCl at pH 8.0, with a final concentration of 0.1 g/mL. Upon high-pressure homogenization-mediated disruption of the suspended cells, the proteins were purified using a Ni-nitrilotriacetate column. The proteins were eluted using a buffer comprising 250 mM NaCl, 250 mM imidazole, 5% glycerol, and 50 mM Tris-HCl pH 8. Imidazole was removed with a desalting column. Protein analyses were performed using sodium dodecyl sulfate polyacrylamide gel electrophoresis. For subsequent enzymatic assays, protein samples were concentrated to 10 mg/mL.

### 2.3 Enzymatic activity determination

The glycosylation specificity and catalytic activity of UGT94D1 or its mutants were assessed by incubating 5 μM of the enzyme with 0.5 mM Reb D in 200 μL reaction solutions containing 50 mM Tris-HCl at pH 8.0, 10 mM MnCl_2_, and 5 mM UDPG. Enzymatic reactions were carried out at 35°C for 4 h, followed by heating at 95°C for 5 min. 400 μL methanol was then added to the system and the mixture was centrifuged at 20,000 *g* for 5 min. The resulting supernatants were filtered and prepared for analysis using ultra-performance liquid chromatography (UPLC) or liquid chromatography-mass spectrometry (LC-MS).

For the determination of kinetic parameters of UGT94D1 toward Reb D, reaction mixtures (200 μL) consisted of 50 mM Tris-HCl pH 8.0, 10 mM MnCl_2_, 5 mM UDPG, varying concentrations of Reb D (0–2 mM), and purified enzyme (1–10 μg). After completing the above operations, analysis was conducted using a Waters Acquity UPLC system at 40°C. The detection wavelength was 210 nm. Elution solvents included acetonitrile (A) and 11.5 mM NaH_2_PO_4_ buffer (pH 2.6, B). A linear gradient elution method was used (0–1 min: 15% A, 6 min: 40% A, and 7–8 min: 15% A) with a flow rate of 0.3 mL/min.

### 2.4 Molecular docking and molecular dynamics simulations

The structural models of UGT94D1 and UDT94D1-F119I-D188P were built using AlphaFold (Jumper et al., 2021), and then UDPG and Reb D were docked into UGT94D1 with the Glide module of Schrödinger 2021. The reasonable conformation of UGT94D1-UDPG-Reb D and UDT94D1-F119I-D188P-UDPG-Reb D with the best score was chosen for subsequent molecular dynamics (MD) simulations. The protein was simulated using the FF14SB force field (Maier et al., 2015), while UDPG and Reb D were modeled using the General Amber force field (Wang et al., 2004; Wang et al., 2006). The protein complex was then solvated with a TIP3P water box (Jorgensen et al., 1983), which has at least 12 Å from the protein to the boundary of the water box. All MD simulations were implemented using the pmemd module of Amber20 (Case, et al., 2020). The procedure of MD simulations was performed as our previous study (Guo et al., 2022; Yang et al., 2023).

### 2.5 Optimization of cascade reaction conditions

Cascade reaction systems (200 μL reaction volumes) were prepared in which the following conditions were varied: pH (5.5–10), sucrose concentration (100–1,000 mM), concentration of UGT94D1-F119I/D188P (1–15 μM), concentration of *At*SuSy (1–15 μM) and reaction temperature (25°C–45°C). The reactions were performed using 50 mM buffer, 2.5 mM Reb D and 0.6 mM UDP and were incubated for 20 min. The reactions were terminated at 95°C for 5 min, followed by the addition of 10 times the volume of methanol, and analysis by UPLC-MS.

### 2.6 Cell-free cascade reaction for the preparation of Reb M2

Under optimal conditions, cell-free cascade reactions (2 mL) were conducted. The reaction system included 13 μM UGT94D1-F119I/D188P, 11 μM *At*SuSy, 50 mM KPI (pH 8.0), 600 mM sucrose, 2.5 mM Reb D and 0.6 mM UDPG. The reactions were performed at 35°C for 8 h. Reb D (50 μL, 100 mM) was added incrementally at different time points (0.5–4.5 h, with 30 min intervals). Samples were taken at different time points (0–5 h with 30 min intervals and 6–8 h with 1 h intervals). The reactions were terminated at 95°C for 5 min, followed by the addition of 20 times the volume of methanol, and analysis by UPLC-MS.

For structure confirmation, the sample was purified with semi prepared HPLC, and the instrument and operative parameters were consistent with our previous work (Ping et al., 2022). The purified sample was further analyzed with nuclear magnetic resonance (NMR) spectroscopy.

## 3 Results and discussion

### 3.1 Selective glycosylation of Reb D to synthesize Reb M2 by UGT94D1

**FIGURE 1:**
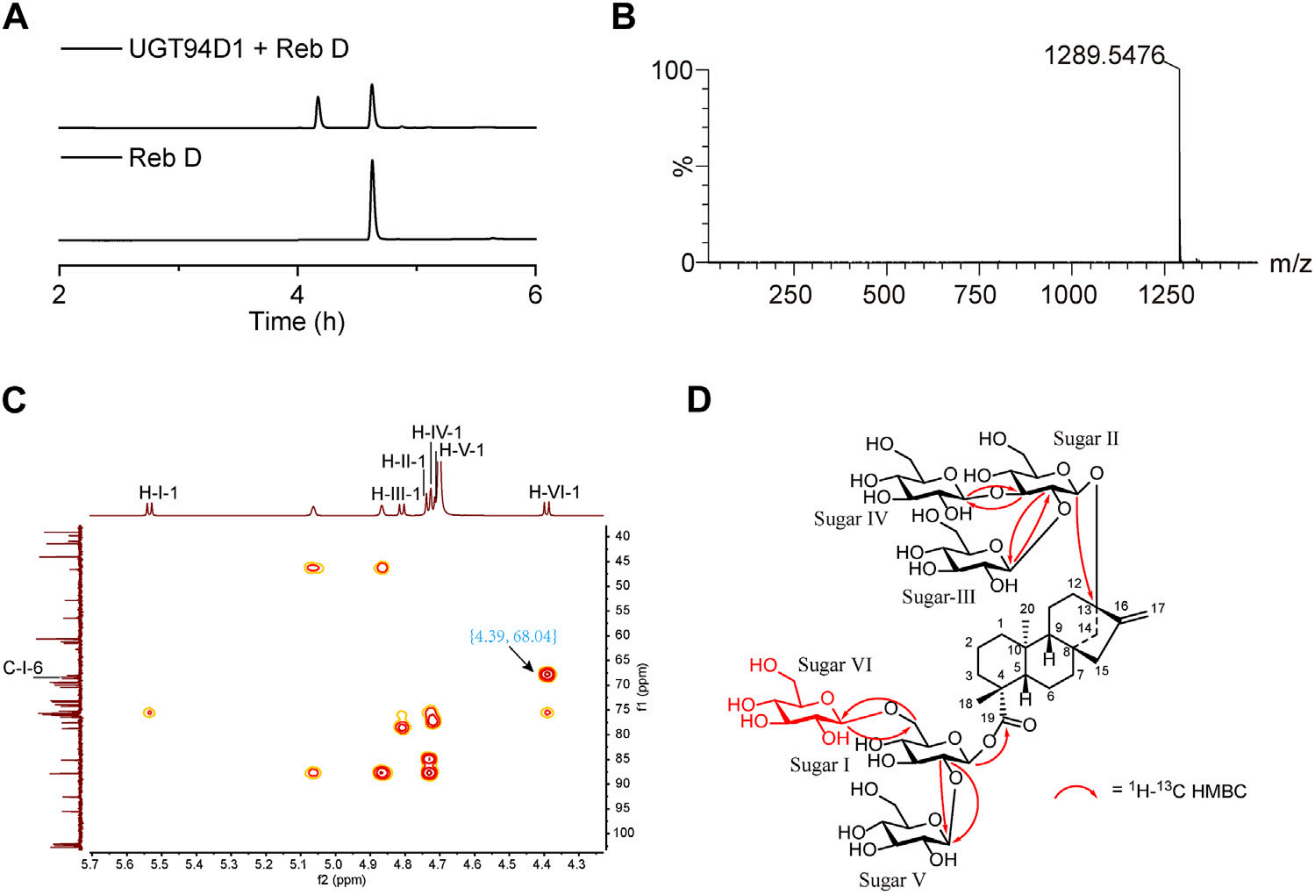
Selective synthesis of Reb M2 from Reb D catalyzed by glycosyltransferase UGT94D1. **(A)** UPLC analysis of the glycosylated product from Reb D by UGT94D1. **(B)** LC-MS analysis of the glycosylated product from Reb D by UGT94D1. **(C)** The correlation between H-1 of new sugar VI (δ_H_ 4.39) and C-6 of sugar Ⅰ (δ_C_ 68.04) in ^1^H-^13^C heteronuclear multiple bond correlation (HMBC) spectrum of Reb M2 (D_2_O). **(D)** ^1^H-^13^C HMBC correlations of Reb M2.

The activity of purified UGT94D1 was evaluated with UDPG as the glycosyl donor (Supplementary Figure S1). UPLC analysis shows the emergence of a single product (Figure 1A). This product was further analyzed by LC-MS (Figure 1B), and a peak at *m/z* 1,289.5476 ([M-H]^-^ ion) was observed. This peak corresponds to a compound with the molecular formula C_56_H_90_O_33_, which is consistent with a mono-glycosylated derivative of Reb D. The structure of this product was further investigated by 1D and 2D NMR spectroscopy (Figures 1C, D; Supplementary Figures S2–S8; Supplementary Table S3), and it was identified as 13-[(2-O-*β*-D-glucopyranosyl-3-O-*β*-D-glucopyranosyl-*β*-D-glucopyranosyl)oxy] ent-kaur-16-en-19-oic acid-[(2-O-*β*-D-glucopyranosyl-6-O-*β*-D-glucopyranosyl-*β*-D-glucopyranosyl) ester] (Figures 1C, D), demonstrating the formation of a 1→6 glycosidic bond attached to the 6-OH of sugar I of Reb D. Our structural characterization data were thus consistent with the structure of Reb M2 as reported in the literature (Prakash, et al., 2014a).

Next, the enzymatic and kinetic properties of UGT94D1 toward Reb D were investigated under the optimal reaction conditions (pH 8.0, 35°C) (Supplementary Figure S9). The *K*_m_ of UGT94D1 toward Reb D was determined to be 0.89 ± 0.05 mM, and the *k*_cat_ was determined to be 0.33 ± 0.08 min^−1^. The resulting relatively low value of *k*_cat_/*K*_m_ suggested the necessity to enhance the catalytic activity of UGT94D1 through protein engineering.

### 3.2 Improving the catalytic activity of UGT94D1 toward Reb D through protein engineering

**FIGURE 2:**
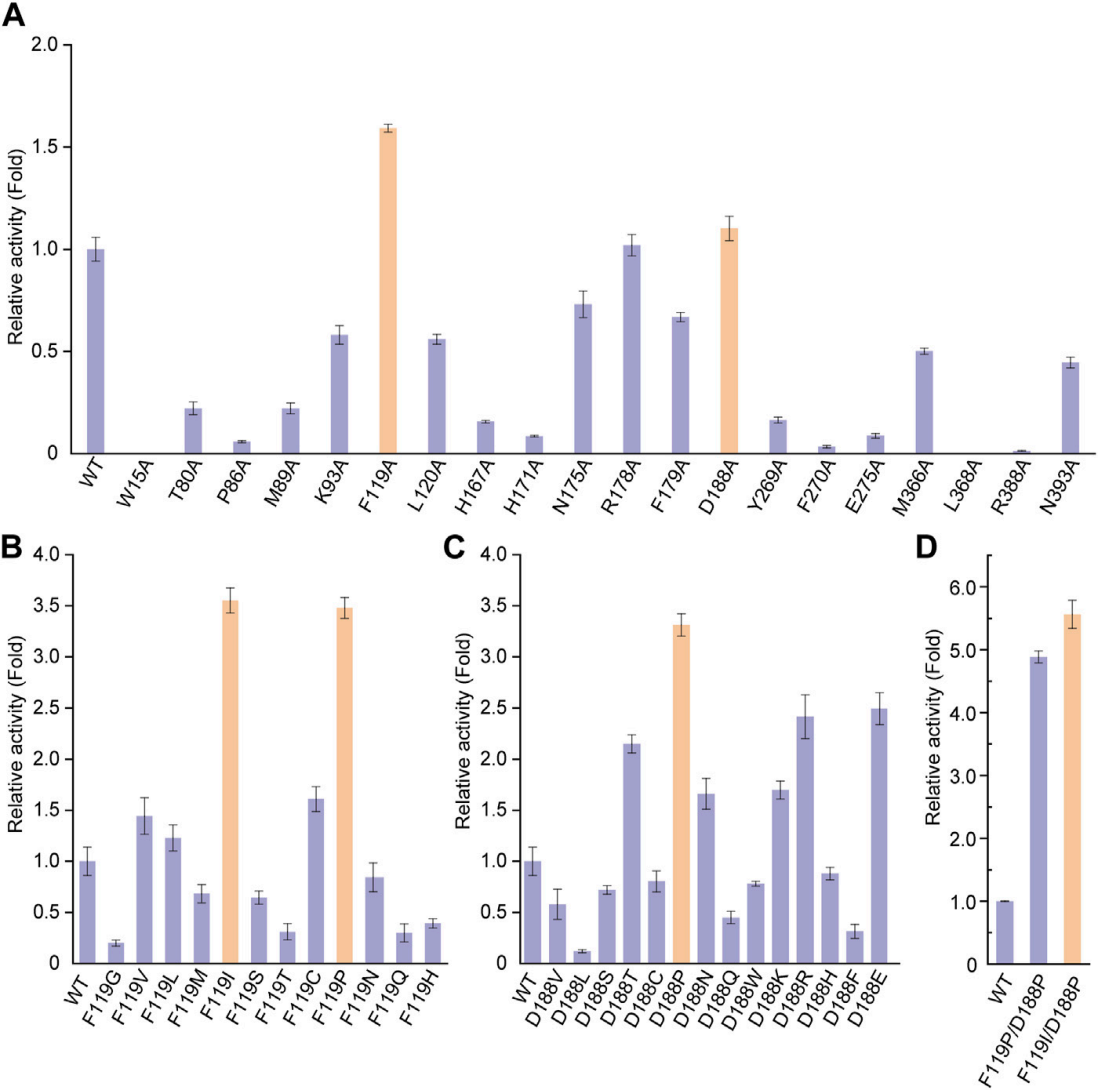
Protein engineering of UGT94D1. **(A)** Relative activities of alanine-scanning mutants of selected residues. Orange columns mean the mutants with improvement in the catalytic activity of UGT94D1. **(B)** Relative activities of different F119 mutants. **(C)** Relative activities of different D188 mutants. **(D)** Relative activities of different combinatorial mutants. UGT94D1 is used as the parent enzyme for mutagenesis, and its activity is set to 1. Orange column means the mutant with the highest catalytic activity. Error bars represent the standard deviation of three duplications.

To improve the catalytic activity of UGT94D1 toward Reb D, structure-guided engineering of UGT94D1 was performed. The structural model of UGT94D1 was first built using AlphaFold (Jumper et al., 2021) and then UDPG and Reb D were docked into the predicted model (Supplementary Figures S10, S11). Comprehensive analysis revealed that the substrate binding pocket of UGT94D1 is located on the surface of the protein, and it features a relatively large cavity for accommodating the sugar moieties. The surrounding amino acids (P86, M89, H171, N175, R178, Y269, E275, M366, L368, H367, R388 and N393) may be involved in hydrophobic interactions with these sugar moieties, thereby facilitating the binding of Reb D (Supplementary Figure S12A). Meanwhile, residue D188 is positioned to form a hydrogen bond interaction with Reb D (Supplementary Figure S12B). In addition to these potentially critical binding residues, other residues (W15, T80, K93, F119, L120, F179, F270) located within 4 Å of the substrate binding pocket (Supplementary Figure S12C) were also analyzed via alanine scanning mutagenesis. As shown in Figure 2A, mutants F119A and D188A exhibited catalytic activities that were enhanced by 1.59- and 1.10-fold, respectively. These two residues were selected for saturation mutagenesis to further enhance the catalytic activity of UGT94D1. We found that mutation of F119 to isoleucine (F119I) and proline (F119P) led to increases of catalytic activity of 3.55- and 3.48-fold, respectively (Figure 2B). In addition, several mutations of D188 were found to increase the catalytic activity. Among them, mutant D188P exhibited the highest catalytic activity, which was increased by 3.31-fold compared to the wild-type (Figure 2C). Upon combinatorial mutagenesis at positions 119 and 188 (Figure 2D), we found that the catalytic activities of mutants F119I/D188P and F119P/D188P were further improved, as these enzymes exhibited catalytic activities that were 6.33 and 4.91 times that of UGT94D1, respectively. Therefore, UGT94D1-F119I/D188P was chosen for the scale-preparation of Reb M2.

### 3.3 Understanding the improved catalytic activity of UGT94D1-F119I/D188P by MD simulations

**FIGURE 3:**
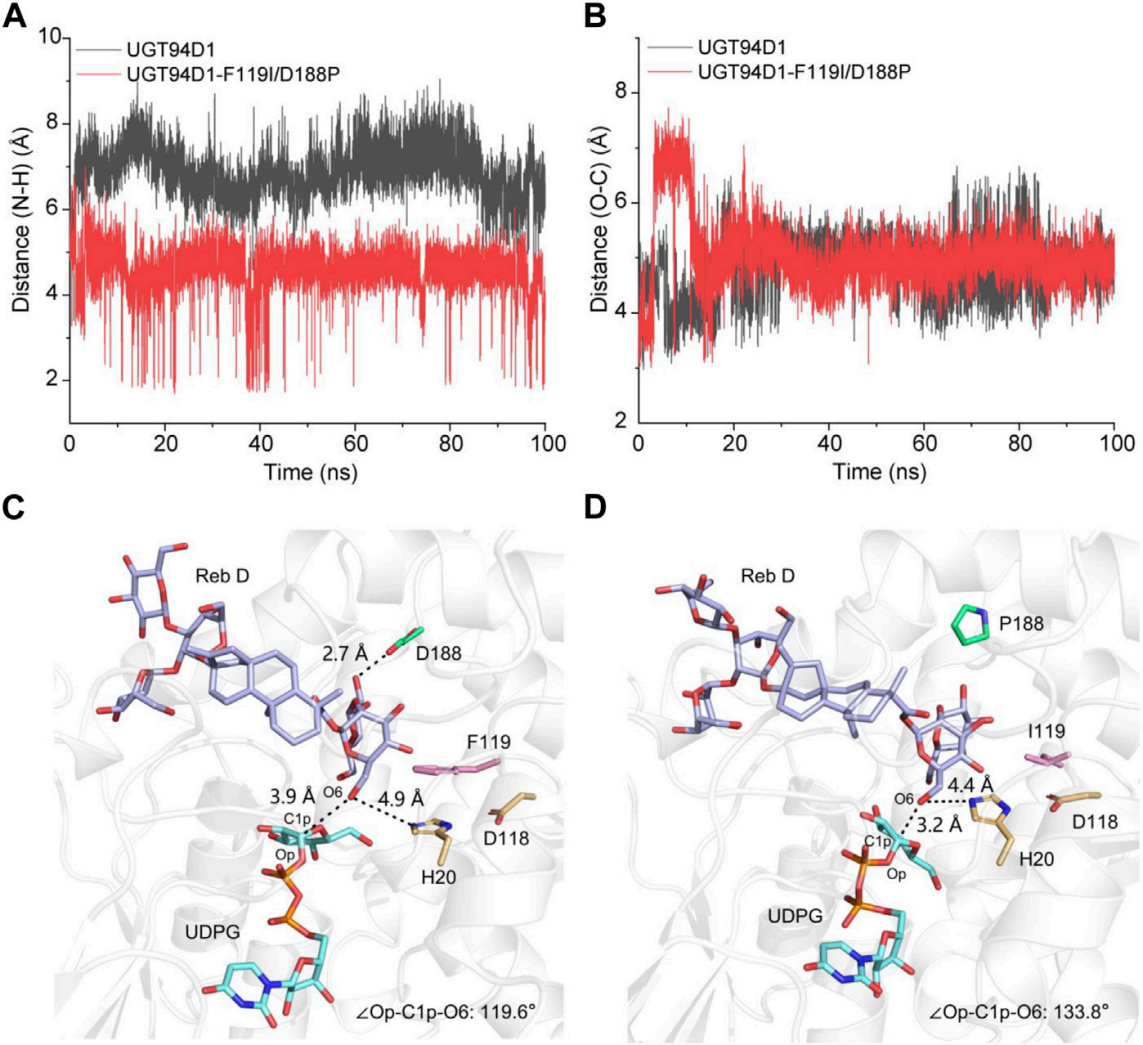
Variations of distance and the representative catalytic conformation of UGT94D1-UDPG-Reb D and UGT94D1-F119I/D188P-UDPG-Reb D in MD stimulations. **(A)** Distance between the nitrogen atom at ε position of catalytic residue H20 and the hydrogen atom of 6-OH of sugar I along 100 ns MD simulations. **(B)** Distance between C1p of UDPG and the hydroxyl oxygen atom in C-6 of sugar I along 100 ns MD simulations. **(C)** Representative catalytic conformation of UGT94D1 in MD simulations. Residues D188 and F119 are represented as green and pink sticks, respectively. The active sites (H20 and D118) are indicated in yellow sticks. **(D)** Representative catalytic conformation of UGT94D1-F119I/D188P in MD simulations. The black dash lines indicate the distances. Residues D188 and F119 are represented as green and pink sticks, respectively. Catalytic sites (H20 and D118), Reb D, and UDPG are shown as yellow, purple, and cyan sticks, respectively. The atoms oxygen and nitrogen are shown in the colors red and blue, respectively.

To study the molecular mechanism behind the catalytic enhancement of UGT94D1-F119I/D188P toward Reb D, we conducted molecular docking and 100 ns unconstrained molecular dynamics (MD) simulations of UGT94D1-F119I/D188P-UDPG-Reb D and UGT94D1-UDPG-Reb D. Based on preliminary mechanism research (Breton et al., 2006; Rahimi et al., 2019; Lin et al., 2020), the distance between the nitrogen atom at ε position of catalytic residue H20 and the hydroxyl hydrogen atom at C-6 of sugar I in Reb D and the distance between C1p of UDPG and the hydroxyl oxygen atom at C-6 of sugar I in Reb D were analyzed (Figures 3A, B). The results revealed that the distance between the Nε of His20 and the H of OH6 was shorter in UGT94D1-F119I/D188P compared to that in UGT94D1 (Figure 3A), while the distance between the electrophile C1p of UDPG and the nucleophilic oxygen of OH6 did not exhibit significant changes (Figure 3B).

To investigate the difference in conformation between UGT94D1-F119I/D188P-UDPG-Reb D and UGT94D1-UDPG-Reb D, representative conformations were obtained through MD simulations (Figures 3C, D). The obtained conformations revealed that mutations to the amino acids at positions 119 and 188 reshaped the hydrophobic binding pocket of the enzyme, making substrate Reb D binding more favorable. As a result, the distance between the nitrogen atom at ε position of catalytic residue H20 and the hydroxyl hydrogen atom at C-6 of sugar I in Reb D decreased from 4.9 to 4.4 Å. In addition, the Op-C1p-O6 angle in the UGT94D1-F119P/D188P (133.8^o^) is closer to the ideal position than that in UGT94D1 (119.6^o^), indicating a more favorable catalytic conformation (Teze et al., 2021). These conformational changes observed are highly favorable for the deprotonation of the hydroxy group at C-6 of sugar I in Reb D, thus improving the catalytic activity of the enzyme.

### 3.4 Cascade reaction for scale-preparation of Reb M2

**FIGURE 4:**
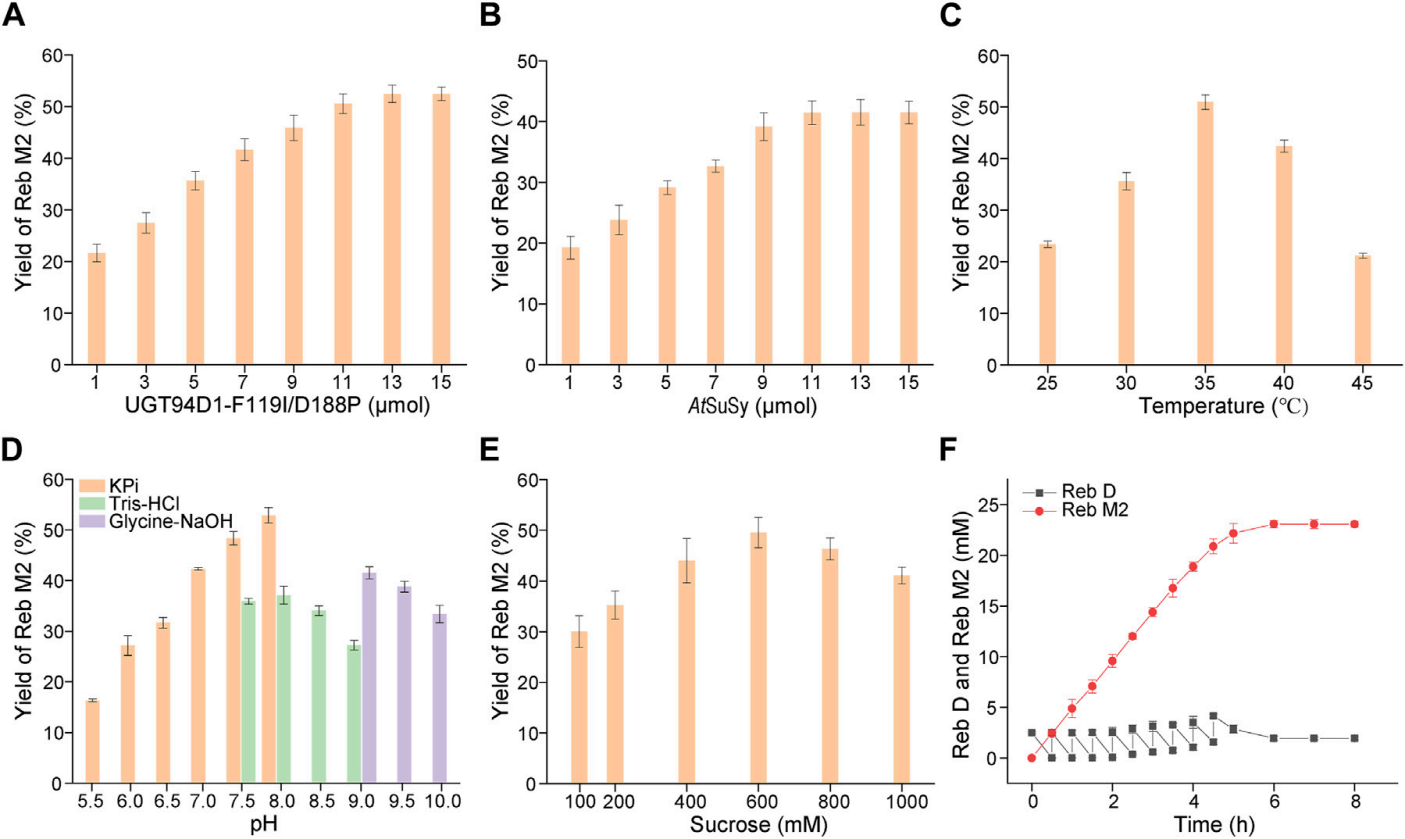
Optimization of the cascade reaction conditions for UGT94D1-F119I/D188P-*At*SuSy. **(A)** Optimization of the concentration of UGT94D1-F119I/D188P. **(B)** Optimization of the concentration of *At*SuSy. **(C)** Temperature optimization. **(D)** pH optimization. **(E)** Optimization of the concentration of sucrose. **(F)** Synthesis of Reb M2 in a cascade reaction with gradual addition of Reb D. The red line corresponds to the concentration of Reb M2, while the black line represents the concentration of Reb D. Error bars represent the standard deviation of three duplications.

To develop a scale-preparation of Reb M2, the variant UGT94D1-F119I/D188P was combined with *At*SuSy from *A. thaliana*, which was utilized to recycle UDPG from UDP using sucrose as a cost-effective sugar donor, to establish a cascade reaction. In order to optimize the production, the concentrations of the two enzymes, UGT94D1-F119I/D188P and *At*SuSy, were first investigated, and the maximum yield of Reb M2 was achieved using 13 μM UGT94D1-F119I/D188P and 11 μM *At*SuSy (Figures 4A, B). Then, the effects of temperature, pH and sucrose concentration were also examined. The optimal conditions for the cascade reaction were determined to be a reaction temperature of 35°C, pH 8.0 and sucrose concentration 600 mM (Figures 4C–E). Notably, higher concentrations of sucrose led to decreased yields of Reb M2. These optimized conditions were employed for a scale-preparation of Reb M2. Considering the low solubility of Reb D, a strategy of gradually adding the substrate Reb D was employed (Figure 4F). It was found that Reb D underwent glycosylation at a rapid rate, with almost all of it being converted to Reb M2 within 5 h. Subsequently, as a result of product inhibition and enzyme activity loss, the production rate of Reb M2 gradually decreased. Ultimately, after 8 h of stepwise addition of Reb D, a total of 23.08 mM (29.79 mg/mL) of Reb M2 was produced, achieving a yield of 92% from the initial concentration of 25 mM (28.23 mg/mL) Reb D in the optimized cascade reaction system.

## 4 Conclusion

In summary, the selective synthesis of a new SG, Reb M2, was achieved using the glycosyltransferase UGT94D1. Through structure-guided evolution, a mutant form of UGT94D1 with significantly enhanced catalytic activity was created; this mutant, UGT94D1-F119I/D188P exhibited a 6.33-fold improvement in catalytic activity as compared to UGT94D1. Molecular dynamics simulations demonstrated that the enhancement of its activity is attributed to the reduction in the distance between the substrate Reb D and the catalytic residues, as well as the increase in the Op-C1p-O6 angle, which favors the deprotonation of the hydroxyl group on Reb D and the formation of the glycosidic bond. By coupling this mutant with sucrose synthase *At*SuSy in a cascade reaction system, efficient production of Reb M2 was realized, and 23.08 mM (29.79 mg/mL) of Reb M2 was generated, representing a yield of 92%. Therefore, this study provides an efficient method for the selective synthesis of Reb M2 to support its applications in various fields.

## Data Availability

The datasets presented in this study can be found in online repositories. The names of the repository/repositories and accession number(s) can be found in the article/Supplementary Material. The accession numbers mentioned in this article include https://www.ncbi.nlm.nih.gov/protein/XP_011076907.1 and https://www.ncbi.nlm.nih.gov/protein/NP_001031915.

## References

[B1] Ahmed J. Preissner S. Dunkel M. Worth C. L. Eckert A. Preissner R. (2011). SuperSweet-a resource on natural and artificial sweetening agents. Nucleic Acids Res. 39, D377–D382. 10.1093/nar/gkq917 20952410 PMC3013782

[B2] Bhardwaj V. Singh R. Singh P. Purohit R. Kumar S. (2020). Elimination of bitter-off taste of stevioside through structure modification and computational interventions. J. Theor. Biol. 486, 110094. 10.1016/j.jtbi.2019.110094 31783061

[B3] Brandt W. Schulze E. Liberman-Aloni R. Bartelt R. Pienkny S. Carmeli-Weissberg M. (2021). Structural modeling of two plant UDP-dependent sugar-sugar glycosyltransferases reveals a conserved glutamic acid residue that is a hallmark for sugar acceptor recognition. J. Struct. Biol. 213, 107777. 10.1016/j.jsb.2021.107777 34391905

[B4] Breton C. Snajdrova L. Jeanneau C. Koca J. Imberty A. (2006). Structures and mechanisms of glycosyltransferases. Glycobiology 16, 29r–37r. 10.1093/glycob/cwj016 16037492

[B5] Caputi L. Malnoy M. Goremykin V. Nikiforova S. Martens S. (2012). A genome-wide phylogenetic reconstruction of family 1 UDP-glycosyltransferases revealed the expansion of the family during the adaptation of plants to life on land. Plant J. 69, 1030–1042. 10.1111/j.1365-313X.2011.04853.x 22077743

[B6] Case D. A. Belfon K. Ben-Shalom I. Y. Brozell S. R. Cerutti D. S. Cheatham T. E. III (2020). Amber 2020. San Francisco, CA: University of California.

[B7] Ceunen S. Geuns J. M. C. (2013). Steviol glycosides: chemical diversity, metabolism, and function. J. Nat. Prod. 76, 1201–1228. 10.1021/np400203b 23713723

[B8] DuBois G. E. Prakash I. (2012). Non-caloric sweeteners, sweetness modulators, and sweetener enhancers. Annu. Rev. Food Sci. Technol. 3, 353–380. 10.1146/annurev-food-022811-101236 22224551

[B9] Gantait S. Das A. Mandal N. (2015). Stevia: a comprehensive review on ethnopharmacological properties and *in vitro* regeneration. Sugar Tech. 17, 95–106. 10.1007/s12355-014-0316-3

[B10] Gerwig G. J. te Poele E. M. Dijkhuizen L. Kamerling J. P. (2016). Stevia glycosides: chemical and enzymatic modifications of their carbohydrate moieties to improve the sweet-tasting quality. Adv. Carbohydr. Chem. Biochem. 73, 1–72. 10.1016/bs.accb.2016.05.001 27816105

[B11] Guo B. D. Deng Z. W. Meng F. Wang Q. F. Zhang Y. Yuan Z. B. (2022). Enhancement of rebaudioside M production by structure-guided engineering of glycosyltransferase UGT76G1. J. Agric. Food Chem. 70, 5088–5094. 10.1021/acs.jafc.2c01209 35417157

[B12] Hellfritsch C. Brockhoff A. Stahler F. Meyerhof W. Hofmnann T. (2012). Human psychometric and taste receptor responses to steviol glycosides. J. Agric. Food Chem. 60, 6782–6793. 10.1021/jf301297n 22616809

[B13] Jorgensen W. L. Chandrasekhar J. Madura J. D. Impey R. W. Klein M. L. (1983). Comparison of simple potential functions for simulating liquid water. J. Chem. Phys. 79, 926–935. 10.1063/1.445869

[B14] Jumper J. Evans R. Pritzel A. Green T. Figurnov M. Ronneberger O. (2021). Highly accurate protein structure prediction with AlphaFold. Nature 596, 583–589. 10.1038/s41586-021-03819-2 34265844 PMC8371605

[B15] Li J. Yang J. G. Mu S. C. Shang N. Liu C. Zhu Y. M. (2020). Efficient O-glycosylation of triterpenes enabled by protein engineering of plant glycosyltransferase UGT74AC1. ACS Catal. 10, 3629–3639. 10.1021/acscatal.9b05232

[B16] Li Y. Chen K. Q. Zhou F. F. Hao N. Ouyang P. K. (2016). A method for the production of Rebaudioside M2 through the enzymatic catalysis of a recombinant bacterium. CN201611142851.5.

[B17] Libik-Konieczny M. Capecka E. Tuleja M. Konieczny R. (2021). Synthesis and production of steviol glycosides: recent research trends and perspectives. Appl. Microbiol. Biotechnol. 105, 3883–3900. 10.1007/s00253-021-11306-x 33914136 PMC8140977

[B18] Lin M. Wang F. Zhu Y. S. (2020). Modeled structure-based computational redesign of a glycosyltransferase for the synthesis of rebaudioside D from rebaudioside A. Biochem. Eng. J. 159, 107626. 10.1016/j.bej.2020.107626

[B19] Lombard V. Ramulu H. G. Drula E. Coutinho P. M. Henrissat B. (2014). The carbohydrate-active enzymes database (CAZy) in 2013. Nucleic Acids Res. 42, D490–D495. 10.1093/nar/gkt1178 24270786 PMC3965031

[B20] Maier J. A. Martinez C. Kasavajhala K. Wickstrom L. Hauser K. E. Simmerling C. (2015). ff14SB: improving the accuracy of protein side chain and backbone parameters from ff99SB. J. Chem. Theory Comput. 11, 3696–3713. 10.1021/acs.jctc.5b00255 26574453 PMC4821407

[B21] Nidetzky B. Gutmann A. Zhong C. (2018). Leloir glycosyltransferases as biocatalysts for chemical production. ACS Catal. 8, 6283–6300. 10.1021/acscatal.8b00710

[B22] Noguchi A. Fukui Y. Iuchi-Okada A. Kakutani S. Satake H. Iwashita T. (2008). Sequential glucosylation of a furofuran lignan, (+)‐sesaminol, by *Sesamum indicum* UGT71A9 and UGT94D1 glucosyltransferases. Plant J. 54, 415–427. 10.1111/j.1365-313X.2008.03428.x 18248594

[B23] Ohtani K. Aikawa Y. Kasai R. Chou W. H. Yamasaki K. Tanaka O. (1992). Minor diterpene glycosides from sweet leaves of rubus-suavissimus. Phytochemistry, 31, 1553–1559. 10.1016/0031-9422(92)83105-8

[B24] Olsson K. Carlsen S. Semmler A. Simon E. Mikkelsen M. D. Moller B. L. (2016). Microbial production of next-generation stevia sweeteners. Microb. Cell Factories 15, 207. 10.1186/s12934-016-0609-1 PMC5142139 27923373

[B25] Ono E. Waki T. Oikawa D. Murata J. Shiraishi A. Toyonaga H. (2020). Glycoside-specific glycosyltransferases catalyze regio-selective sequential glucosylations for a sesame lignan, sesaminol triglucoside. Plant J. 101, 1221–1233. 10.1111/tpj.14586 31654577

[B26] Paquette S. Møller B. L. Bak S. (2003). On the origin of family 1 plant glycosyltransferases. Phytochemistry 62, 399–413. 10.1016/S0031-9422(02)00558-7 12620353

[B27] Perrier J. D. Mihalov J. J. Carlson S. J. (2018). FDA regulatory approach to steviol glycosides. Food Chem. Toxicol. 122, 132–142. 10.1016/j.fct.2018.09.062 30268795

[B28] Ping Q. Yang L. F. Jiang J. J. Yuan J. C. Ai S. Sun S. Q. (2022). Efficient synthesis of rebaudioside D2 through UGT94D1-catalyzed regio-selective glycosylation. Carbohydr. Res. 522, 108687. 10.1016/j.carres.2022.108687 36270051

[B29] Prakash I. Bunders C. Devkota K. P. Charan R. D. Ramirez C. Priedemann C. (2014a). Isolation and characterization of a novel rebaudioside M isomer from a bioconversion reaction of rebaudioside A and NMR comparison studies of rebaudioside M isolated from Stevia rebaudiana Bertoni and Stevia rebaudiana Morita. Biomolecules 4, 374–389. 10.3390/biom4020374 24970220 PMC4101487

[B30] Prakash I. Chaturvedula V. S. Markosyan A. (2014b). Structural characterization of the degradation products of a minor natural sweet diterpene glycoside Rebaudioside M under acidic conditions. Int. J. Mol. Sci. 15, 1014–1025. 10.3390/ijms15011014 24424316 PMC3907853

[B31] Purkayastha S. Markosyan A. Prakash I. Bhusari S. Pugh G. Lynch B. (2016). Steviol glycosides in purified stevia leaf extract sharing the same metabolic fate. Regul. Toxicol. Pharmacol. 77, 125–133. 10.1016/j.yrtph.2016.02.015 26924787

[B32] Rahimi S. Kim J. Mijakovic I. Jung K. H. Choi G. Kim S. C. (2019). Triterpenoid-biosynthetic UDP-glycosyltransferases from plants. Biotechnol. Adv. 37, 107394. 10.1016/j.biotechadv.2019.04.016 31078628

[B33] Schmölzer K. Gutmann A. Diricks M. Desmet T. Nidetzky B. (2016). Sucrose synthase: a unique glycosyltransferase for biocatalytic glycosylation process development. Biotechnol. Adv. 34, 88–111. 10.1016/j.biotechadv.2015.11.003 26657050

[B34] te Poele E. M. Devlamynck T. Jager M. Gerwig G. J. Van de Walle D. Dewettinck K. (2018). Glucansucrase (mutant) enzymes from Lactobacillus reuteri 180 efficiently transglucosylate Stevia component rebaudioside A, resulting in a superior taste. Sci. Rep. 8, 1516. 10.1038/s41598-018-19622-5 29367749 PMC5784128

[B35] Teze D. Coines J. Fredslund F. Dubey K. D. Bidart G. N. Adams P. D. (2021). O-/N-/S-Specificity in glycosyltransferase catalysis: from mechanistic understanding to engineering. ACS Catal. 11, 1810–1815. 10.1021/acscatal.0c04171

[B36] Tian X. Y. Zhong F. Xia Y. X. (2022). Dynamic characteristics of sweetness and bitterness and their correlation with chemical structures for six steviol glycosides. Food Res. Int. 151, 110848. 10.1016/j.foodres.2021.110848 34980386

[B37] Wang J. M. Wang W. Kollman P. A. Case D. A. (2006). Automatic atom type and bond type perception in molecular mechanical calculations. J. Mol. Graph. Model. 25, 247–260. 10.1016/j.jmgm.2005.12.005 16458552

[B38] Wang J. M. Wolf R. M. Caldwell J. W. Kollman P. A. Case D. A. (2004). Development and testing of a general amber force field. J. Comput. Chem., 25, 1157–1174. 10.1002/jcc.20035 15116359

[B39] Wen C. Huang W. Zhu X. L. Li X. S. Zhang F. Jiang R. W. (2018). UGT74AN1, a permissive glycosyltransferase from *Asclepias curassavica* for the regiospecific steroid 3-O-glycosylation. Org. Lett. 20, 534–537. 10.1021/acs.orglett.7b03619 29363978

[B40] Yang S. Hou X. D. Deng Z. W. Yang L. F. Ping Q. Yuan Z. B. (2023). Improving the thermostability of glycosyltransferase YojK by targeting mutagenesis for highly efficient biosynthesis of rebaudioside D. Mol. Catal. 535, 112898. 10.1016/j.mcat.2022.112898

[B41] Yonekura-Sakakibara K. Hanada K. (2011). An evolutionary view of functional diversity in family 1 glycosyltransferases. Plant J. 66, 182–193. 10.1111/j.1365-313X.2011.04493.x 21443631

[B42] Zhang P. Zhang Z. Zhang L. J. Wang J. J. Wu C. S. (2020). Glycosyltransferase GT1 family: phylogenetic distribution, substrates coverage, and representative structural features. Comput. Struct. Biotechnol. J. 18, 1383–1390. 10.1016/j.csbj.2020.06.003 32637037 PMC7316871

