# Supplementary material for: Selective synthesis of rebaudioside M2 through structure-guided engineering of glycosyltransferase UGT94D1
Source: Front Bioeng Biotechnol. 2024 Feb 5;12:1334427. doi: 10.3389/fbioe.2024.1334427 (PMC10875103; doi:10.3389/fbioe.2024.1334427)
Supplement: Supplementary file 1 [file DataSheet1.docx]

Supplementary Material

**Supplementary Table 1** Strains and plasmids.

| **Strains or Plasmids** | **Description** | **Sources** |
| --- | --- | --- |
| **Strains** |  |  |
| Top10 | wild type | Novagen |
| BL21(DE3) | wild type | Novagen |
| BL21- UGT94D1/(mutants) | *E.coli* BL21(DE3) harboring plasmid pET-21b(+)-*UGT94D1*/(mutants) | this study |
| BL21-*At*SuSy | *E.coli* BL21(DE3) harboring plasmid pET-21b(+)-*AtSuSy* | this study |
| **Plasmids** |  |  |
| pET-21b(+)-UGT94D1/(mutants) | pET-21b(+) carrying a glycosyltransferase *UGT94D1* gene from *Sesamum indicum*, Amp^R^ | this study |
| pET-21b(+)-*At*SuSy | pET-21b(+) carrying a sucrose synthase *AtSuSy* gene from from *Arabidopsis thaliana*, Amp^R^ | this study |

**Supplementary Table 2** Primers used for UGT94D1 mutagenesis.

| **Name** | **Sequence (5’-3’)** |
| --- | --- |
| W15A_F | CCGGCTCTTGCTCACGGCCATATCTCC |
| W15A_R | CCGTGAGCAAGAGCCGGAAACATTAAAATGCGGATCGAAC |
| T80A_F | CATGCTACCAACGGCCTGCCAC |
| T80A_R | GCCGTTGGTAGCATGATATGGAGGCGGAAGGATC |
| P86A_F | CCAGCTCACTTGATGTCTACTTTAAAGCGTGC |
| P86A_R | GACATCAAGTGAGCTGGCAGGCCGTTGGTTG |
| M89A_F | TTGGCTTCTACTTTAAAGCGTGCTTTAGATTCAGC |
| M89A_R | TTTAAAGTAGAAGCCAAGTGGGGTGGCAGGC |
| K93A_F | TTAGCTCGTGCTTTAGATTCAGCGCG |
| K93A_R | TCTAAAGCACGAGCTAAAGTAGACATCAAGTGGGGTGGC |
| F119A_F | GATGCTTTACAAAGCTGGGCCAGTGAAG |
| F119A_R | CAGCTTTGTAAAGCATCATATAACACAAGGTCAGGCTTCAG |
| L120A_F | TTTGCTCAAAGCTGGGCCAGTGAAG |
| L120A_R | GCCCAGCTTTGAGCAAAATCATATAACACAAGGTCAGGCTTCAG |
| H171A_F | GAAGCTGAATACGATAACTTCTGTCGTTTTAAAAGTTC |
| H171A_R | TTATCGTATTCAGCTTCACGAAAATAAATAGCAGGGAAAGGG |
| N175A_F | GATGCTTTCTGTCGTTTTAAAAGTTCCGACTCAG |
| N175A_R | AAACGACAGAAAGCATCGTATTCATGTTCACGAAAATAAATAGCAG |
| R178A_F | GTGCTTTTAAAAGTTCCGACTCAGGCACTTC |
| R178A_R | AACTTTTAAAAGCACAGAAGTTATCGTATTCATGTTCACGAAAAT |
| F179A_F | GTCGTGCTAAAAGTTCCGACTCAGGCACTTC |
| F179A_R | AACTTTTAGCACGACAGAAGTTATCGTATTCATGTTCACGAAAAT |
| D188A_F | TCCGCTCAGCTTCGTGTGTCAGATTGC |
| D188A_R | ACACGAAGCTGAGCGGAAGTGCCTGAGTCGGAAC |
| Y269A_F | GAGGCTTTCCTGTCAGCCAATGAAATCGAG |
| Y269A_R | GCTGACAGGAAAGCCTCACTCCCAAATGATGAAAAAACGG |
| F270A_F | TATGCTCTGTCAGCCAATGAAATCGAGG |
| F270A_R | TTGGCTGACAGAGCATACTCACTCCCAAATGATGAAAAAACG |
| E275A_F | AATGCTATCGAGGAAATCGCTTATGGGC |
| E275A_R | ATTTCCTCGATAGCATTGGCTGACAGGAAATACTCACTC |
| M366A_F | CCTGCTCACTTGGATCAACCCTTCAATGCTC |
| M366A_R | TGATCCAAGTGAGCAGGCACAGCAATAATTGGGAC |
| H367A_F | ATGGCTTTGGATCAACCCTTCAATGCTCG |
| H367A_R | GGTTGATCCAAAGCCATAGGCACAGCAATAATTGGGAC |
| L368A_F | CACGCTGATCAACCCTTCAATGCTCGC |
| L368A_R | AGGGTTGATCAGCGTGCATAGGCACAGCAATAATTGG |
| R388A_F | GTTGCTTCACGTCAGGGTAATTTGGATCG |
| R388A_R | CCCTGACGTGAAGCAACGACTTCTTCTCCGAAACCG |
| N393A_F | GGTGCTTTGGATCGTGGAGAGGTTGCTC |
| N393A_R | CCACGATCCAAAGCACCCTGACGTGAGCGAAC |
| F119G_F | GATGGCTTACAAAGCTGGGCCAGTGAAG |
| F119G_R | CAGCTTTGTAAGCCATCATATAACACAAGGTCAGGCTTCAG |
| F119V_F | GATGTCTTACAAAGCTGGGCCAGTGAAG |
| F119V_R | CAGCTTTGTAAGACATCATATAACACAAGGTCAGGCTTCAG |
| F119L_F | GATTTGTTACAAAGCTGGGCCAGTGAAG |
| F119L_R | CAGCTTTGTAACAAATCATATAACACAAGGTCAGGCTTCAG |
| F119M_F | GATATGTTACAAAGCTGGGCCAGTGAAG |
| F119M_R | CAGCTTTGTAACATATCATATAACACAAGGTCAGGCTTCAG |
| F119I_F | GATATTTTACAAAGCTGGGCCAGTGAAG |
| F119I_R | CAGCTTTGTAAAATATCATATAACACAAGGTCAGGCTTCAG |
| F119S_F | GATTCATTACAAAGCTGGGCCAGTGAAG |
| F119S_R | CAGCTTTGTAATGAATCATATAACACAAGGTCAGGCTTCAG |
| F119T_F | GATACGTTACAAAGCTGGGCCAGTGAAG |
| F119T_R | CAGCTTTGTAACGTATCATATAACACAAGGTCAGGCTTCAG |
| F119C_F | GATTGCTTACAAAGCTGGGCCAGTGAAG |
| F119C_R | CAGCTTTGTAAGCAATCATATAACACAAGGTCAGGCTTCAG |
| F119P_F | GATCCCTTACAAAGCTGGGCCAGTGAAG |
| F119P_R | CAGCTTTGTAAGGGATCATATAACACAAGGTCAGGCTTCAG |
| F119N_F | GATAATTTACAAAGCTGGGCCAGTGAAG |
| F119N_R | CAGCTTTGTAAATTATCATATAACACAAGGTCAGGCTTCAG |
| F119Q_F | GATCAATTACAAAGCTGGGCCAGTGAAG |
| F119Q_R | CAGCTTTGTAATTGATCATATAACACAAGGTCAGGCTTCAG |
| F119H_F | GATCACTTACAAAGCTGGGCCAGTGAAG |
| F119H_R | CAGCTTTGTAAGTGATCATATAACACAAGGTCAGGCTTCAG |
| D188V_F | TCCGTCCAGCTTCGTGTGTCAGATTGC |
| D188V_R | ACACGAAGCTGGACGGAAGTGCCTGAGTCGGAAC |
| D188L_F | TCCTTGCAGCTTCGTGTGTCAGATTGC |
| D188L_R | ACACGAAGCTGCAAGGAAGTGCCTGAGTCGGAAC |
| D188S_F | TCCTCACAGCTTCGTGTGTCAGATTGC |
| D188S_R | ACACGAAGCTGTGAGGAAGTGCCTGAGTCGGAAC |
| D188T_F | TCCACGCAGCTTCGTGTGTCAGATTGC |
| D188T_R | ACACGAAGCTGCGTGGAAGTGCCTGAGTCGGAAC |
| D188C_F | TCCTGCCAGCTTCGTGTGTCAGATTGC |
| D188C_R | ACACGAAGCTGGCAGGAAGTGCCTGAGTCGGAAC |
| D188P_F | TCCCCCCAGCTTCGTGTGTCAGATTGC |
| D188P_R | ACACGAAGCTGGGGGGAAGTGCCTGAGTCGGAAC |
| D188N_F | TCCAATCAGCTTCGTGTGTCAGATTGC |
| D188N_R | ACACGAAGCTGATTGGAAGTGCCTGAGTCGGAAC |
| D188Q_F | TCCCAACAGCTTCGTGTGTCAGATTGC |
| D188Q_R | ACACGAAGCTGTTGGGAAGTGCCTGAGTCGGAAC |
| D188W_F | TCCTGGCAGCTTCGTGTGTCAGATTGC |
| D188W_R | ACACGAAGCTGCCAGGAAGTGCCTGAGTCGGAAC |
| D188K_F | TCCAAGCAGCTTCGTGTGTCAGATTGC |
| D188K_R | ACACGAAGCTGCTTGGAAGTGCCTGAGTCGGAAC |
| D188R_F | TCCCGCCAGCTTCGTGTGTCAGATTGC |
| D188R_R | ACACGAAGCTGGCGGGAAGTGCCTGAGTCGGAAC |
| D188H_F | TCCCACCAGCTTCGTGTGTCAGATTGC |
| D188H_R | ACACGAAGCTGGTGGGAAGTGCCTGAGTCGGAAC |
| D188F_F | TCCTTCCAGCTTCGTGTGTCAGATTGC |
| D188F_R | ACACGAAGCTGGAAGGAAGTGCCTGAGTCGGAAC |
| D188E_F | TCCGAGCAGCTTCGTGTGTCAGATTGC |
| D188E_R | ACACGAAGCTGCTCGGAAGTGCCTGAGTCGGAAC |
| F119I D188P_F | TCCCCCCAGCTTCGTGTGTCAGATTGC |
| F119I D188P_R | ACACGAAGCTGGGGGGAAGTGCCTGAGTCGGAAC |
| F119P D188P_F | TCCCCCCAGCTTCGTGTGTCAGATTGC |
| F119P D188P_R | ACACGAAGCTGGGGGGAAGTGCCTGAGTCGGAAC |

^a^A: alanine (Ala); P: Proline (Pro); G: glycine (Gly); K: lysine (Lys); V: Valine (Val); I: isoleucine (Ile); F: phenylalanine (Phe); D: Aspartic acid (Asp); N: Asparagine (Asn); L: leucine (Leu); M: methionine (Met); T: threonine (Thr); E: glutamic acid (Glu); S: serine (Ser)

**Supplementary Table 3** ^1^H and ^13^C NMR (600 and 151 MHz, D_2_O) data assignments of Reb M2.

| Position | Synthesized in this study | | | Reb M2 in the Reference^1^ | |
| --- | --- | --- | --- | --- | --- |
|  | δ_C_ | δ_H_ (*J* in Hz) | HMBC | δ_C_ | δ_H_ (*J* in Hz) |
| 1 | 39.10 | 0.81 (m) |  | 41.9 | 0.93 (m) |
|  |  | 1.81 (m) |  |  | 1.93 (m) |
| 2 | 18.98 | 1.37 (m) |  | 21.8 | 1.49 (m) |
|  |  | 1.77 (m) |  |  | 1.86 (m) |
| 3 | 36.75 | 1.03 (m) |  | 39.8 | 1.16 (m) |
|  |  | 2.18 (d, 13.2) |  |  | 2.28 (d, 13.4) |
| 4 | 40.98 |  |  | 43.7 |  |
| 5 | 56.46 | 1.11 (d, 12.4) |  | 59.2 | 1.24 (d, 12.1) |
| 6 | 21.55 | 1.62 (m) |  | 24.4 | 1.73 (m) |
|  |  | 1.83 (m) |  |  | 1.94 (m) |
| 7 | 41.37 | 1.38 (m) |  | 44.2 | 1.49 (m) |
|  |  | 1.44 (m) |  |  | 1.56 (m) |
| 8 | 44.00 |  |  | 46.9 |  |
| 9 | 52.83 | 0.97 (d, 8.0) |  | 55.5 | 1.09 (d, 7.7) |
| 10 | 39.75 |  |  | 42.4 |  |
| 11 | 19.83 | 1.52 (m) |  | 22.6 | 1.66 (m) |
|  |  | 1.57 (m) |  |  | 1.70 (m) |
| 12 | 36.92 | 1.48 (m) |  | 39.9 | 1.60 (m) |
|  |  | 1.89 (m) |  |  | 2.00 (m) |
| 13 | 87.89 |  |  | 90.9 |  |
| 14 | 44.05 | 1.41 (m) |  | 46.9 | 1.53 (d, 12.6) |
|  |  | 2.12 (m) |  |  | 2.21 (d, 13.6) |
| 15 | 46.60 | 2.03 (d, 17.4) |  | 49.4 | 2.15 (d, 17.2) |
|  |  | 2.10 (m) |  |  | 2.18 (d, 18.1) |
| 16 | 153.08 |  |  | 164.0 |  |
| 17 | 104.27 | 4.86 (s) |  | 107.1 | 4.98 (s) |
|  |  | 5.06 (s) |  |  | 5.6 (s) |
| 18 | 28.20 | 1.18 (3H, s) |  | 31.0 | 1.29 (s) |
| 19 | 178.43 |  |  | 181.5 |  |
| 20 | 16.17 | 0.81 (3H, s) |  | 19.1 | 0.92 (s) |
| Ⅰ-1 | 92.63 | 5.53 (d, 7.9) | 178.43 (C-19) | 95.5 | 5.65 (d, 7.6) |
| Ⅰ-2 | 77.58 | 3.83 (m) | 102.46 (C-V-1) | 80.5 | 3.96 (m) |
| Ⅰ-3 | 76.15 | 3.76 (m) |  | 79.0 | 3.89 (m) |
| Ⅰ-4 | 68.68 | 3.58 (m) |  | 71.5 | 3.71 (m) |
| Ⅰ-5 | 76.46 | 3.60 (m) |  | 79.0 | 3.73 (m) |
| Ⅰ-6 | 68.04 | 3.89 (m) | 102.78 (C-Ⅵ-1) | 70.9 | 4.00 (m) |
|  |  | 4.03 (m) |  |  | 4.15 (d,11.7) |
| Ⅱ-1 | 95.56 | 4.74 (d, 7.6) | 87.89 (C-13) | 98.4 | 4.85 (d, 7.8) |
| Ⅱ-2 | 78.78 | 3.62 (m) | 102.04 (C-Ⅲ-1) | 81.7 | 3.75 (m) |
| Ⅱ-3 | 85.17 | 3.87 (m) | 102.16 (C-Ⅳ-1) | 88.0 | 3.98 (m) |
| Ⅱ-4 | 68.37 | 3.43 (m) |  | 71.3 | 3.54 (m) |
| Ⅱ-5 | 77.58 | 3.84 (m) |  | 80.5 | 3.96 (m) |
| Ⅱ-6 | 60.66 | 3.32 (m) |  | 63.6 | 3.45 (m) |
| Ⅱ-6 |  | 3.65 (m) |  |  | 3.77 (m) |
| Ⅲ-1 | 102.04 | 4.81 (d, 7.9) | 78.78 (C-Ⅱ-2) | 104.9 | 4.92 (d, 7.9) |
| Ⅲ-2 | 73.38 | 3.20 (m) |  | 76.3 | 3.32 (m) |
| Ⅲ-3 | 75.81 | 3.40 (m) |  | 78.8 | 3.51 (m) |
| Ⅲ-4 | 70.44 | 3.14 (m) |  | 73.3 | 3.26 (t, 9.5) |
| Ⅲ-5 | 75.94 | 3.31 (m) |  | 78.8 | 3.44 (m) |
| Ⅲ-6 | 61.27 | 3.62 (m) |  | 64.4 | 3.75 (m) |
|  |  | 3.81 (m) |  |  | 3.94 (m) |
| Ⅳ-1 | 102.16 | 4.73 (d, 7.8) | 85.18 (C-Ⅱ-3) | 105.0 | 4.84 (d, 7.8) |
| Ⅳ-2 | 73.15 | 3.28 (m) |  | 76.1 | 3.41 (m) |
| Ⅳ-3 | 75.96 | 3.35 (m) |  | 78.8 | 3.46 (m) |
| Ⅳ-4 | 69.56 | 3.32 (m) |  | 72.5 | 3.45 (m) |
| Ⅳ-5 | 78.78 | 3.63 (m) |  | 81.7 | 3.75 (m) |
| Ⅳ-6 | 61.54 | 3.44 (m) |  | 65.8 | 3.55 (m) |
|  |  | 3.67 (m) |  |  | 3.78 (m) |
| Ⅴ-1 | 102.46 | 4.72 (d, 7.9) | 77.58 (C-Ⅰ-2) | 105.3 | 4.83 (d, 8.0) |
| Ⅴ-2 | 75.26 | 3.21 (m) |  | 78.5 | 3.32 (m) |
| Ⅴ-3 | 75.67 | 3.41 (m) |  | 78.7 | 3.51 (m) |
| Ⅴ-4 | 70.02 | 3.25 (m) |  | 72.9 | 3.38 (m) |
| Ⅴ-5 | 76.00 | 3.45 (m) |  | 78.8 | 3.55 (m) |
| Ⅴ-6 | 60.66 | 3.64 (m) |  | 63.6 | 3.76 (m) |
|  |  | 3.86 (m) |  |  | 3.97 (m) |
| VI-1 | 102.78 | 4.39 (d, 7.9) | 68.04 (C-I-6) | 105.7 | 4.50 (d, 7.9) |
| VI-2 | 74.27 | 3.24 (m) |  | 78.1 | 3.33 (m) |
| VI-3 | 75.61 | 3.38 (m) |  | 78.6 | 3.49 (m) |
| VI-4 | 69.56 | 3.34 (m) |  | 72.3 | 3.45 (m) |
| VI-5 | 76.09 | 3.37 (m) |  | 78.8 | 3.48 (m) |
| VI-6 | 60.74 | 3.79 (m) |  | 64.1 | 3.92 (m) |
|  |  | 3.83 (m) |  |  | 3.94 (m) |


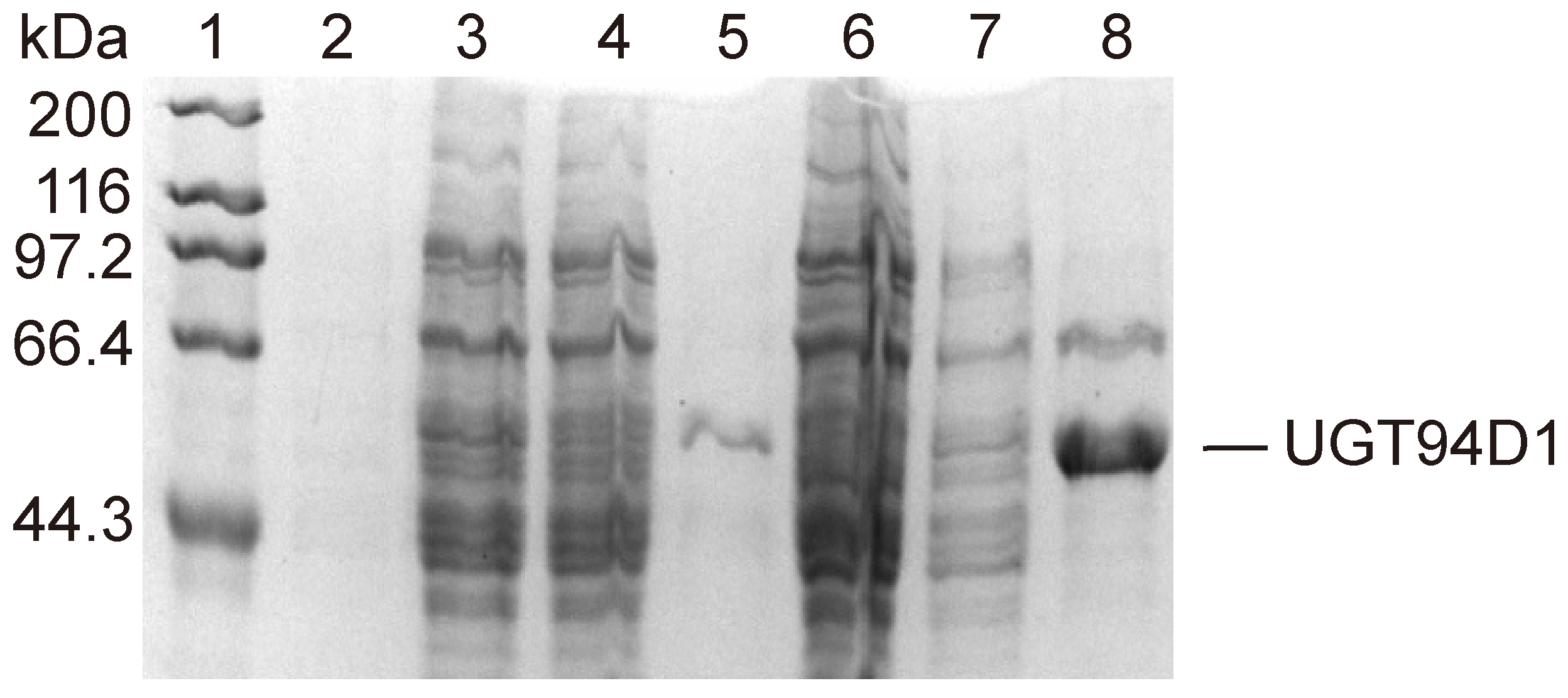


**Supplementary Figure 1.** SDS-PAGE analysis of the heterologous expression of UGT94D1 in *E. coli* BL21(DE3). Lane 1: marker; Lane 2: sample without IPTG induction; Lane 3: crude enzyme; Lane 4: supernatant of crude enzyme; Lane 5: pellet; Lane 6: flow-through fraction; Lane 7: washing fraction; Lane 8: elution faction.


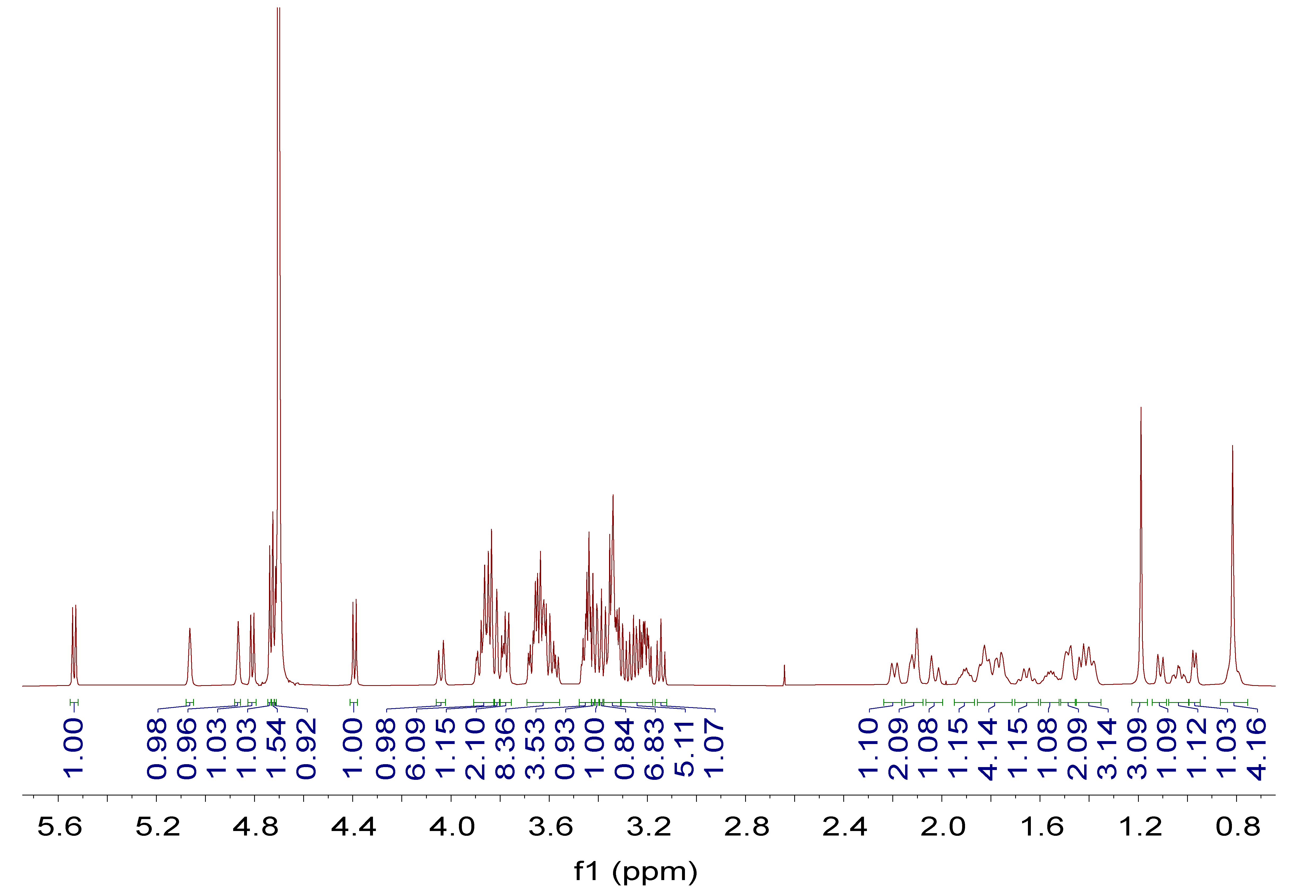


**Supplementary Figure 2.** ^1^H NMR spectrum of Reb M2 (600 MHz, D_2_O).


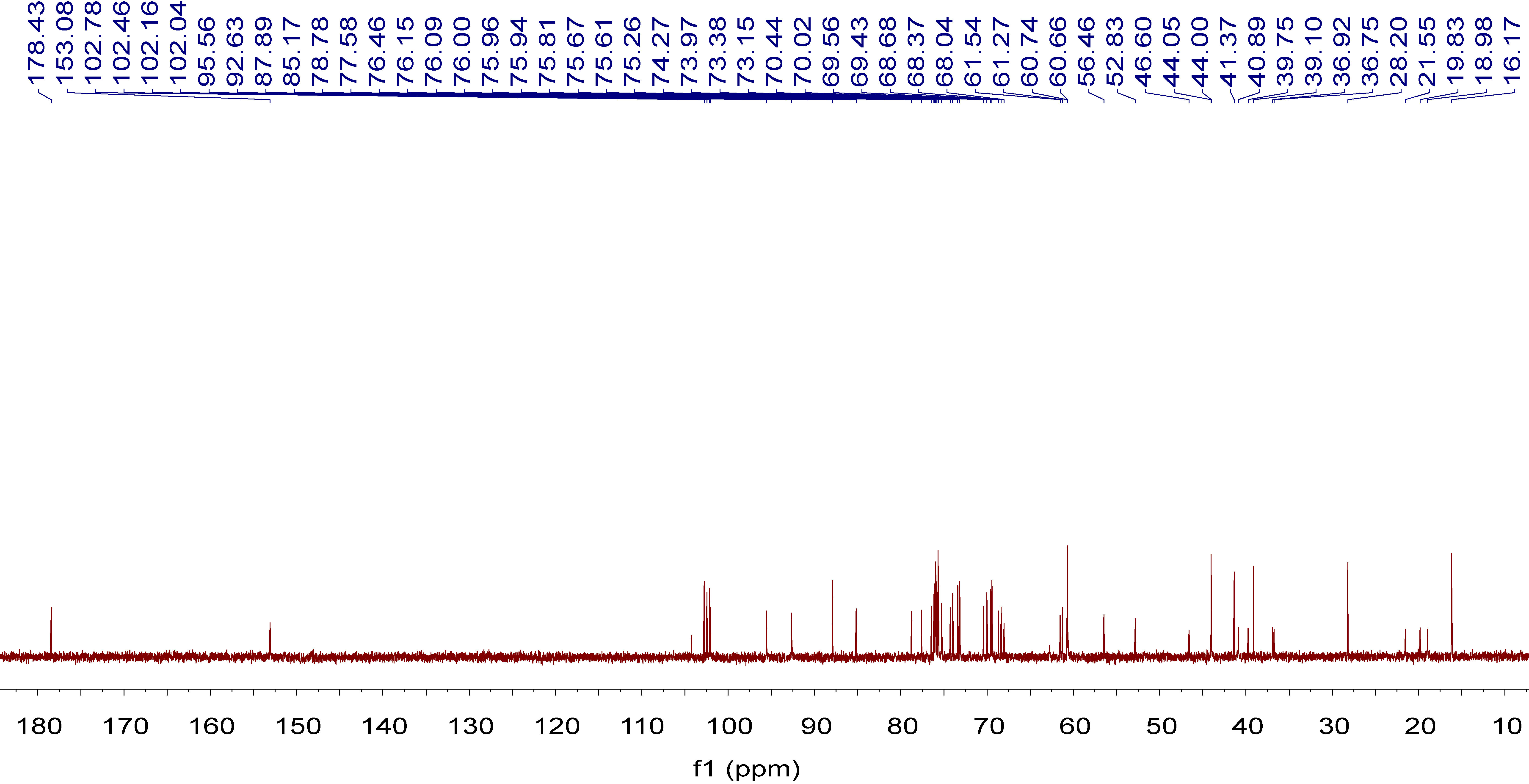


**Supplementary Figure 3.** ^13^C NMR spectrum of Reb M2 (151 MHz, D_2_O).


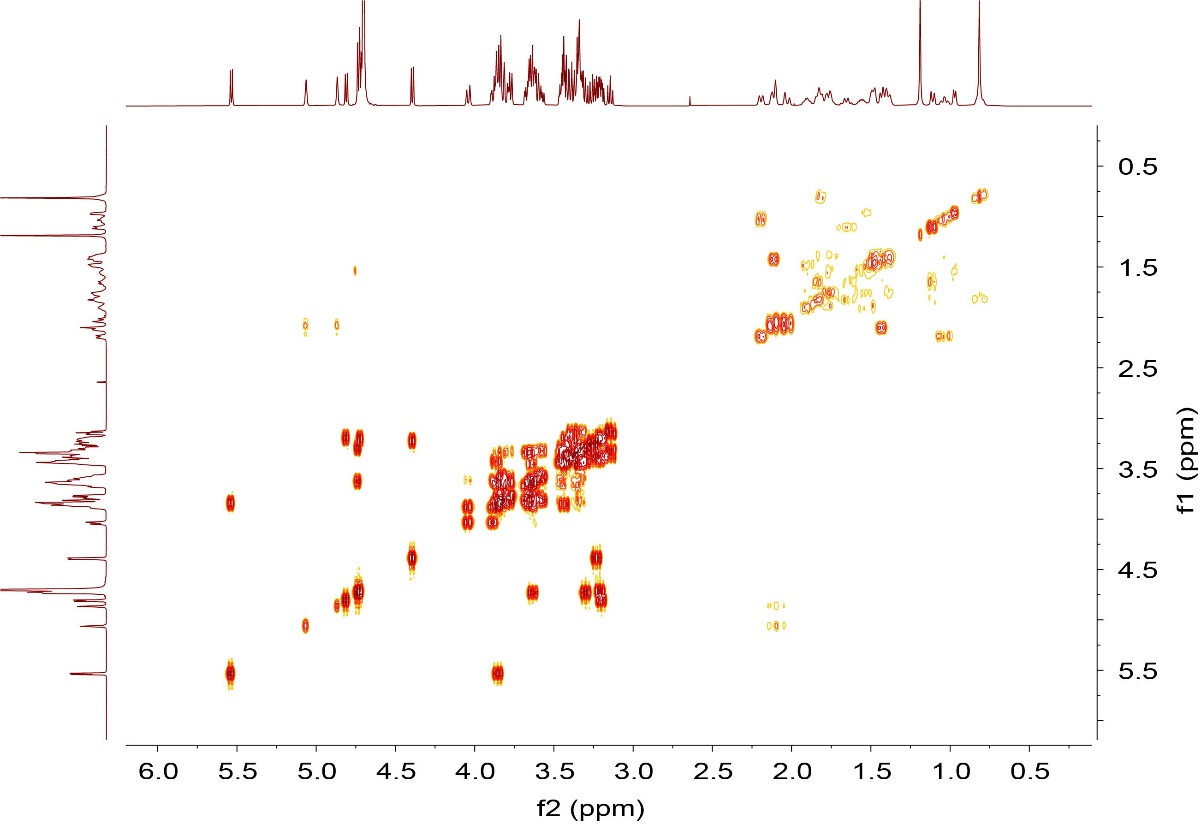


**Supplementary Figure 4.** ^1^H-^1^H COSY spectrum of Reb M2.


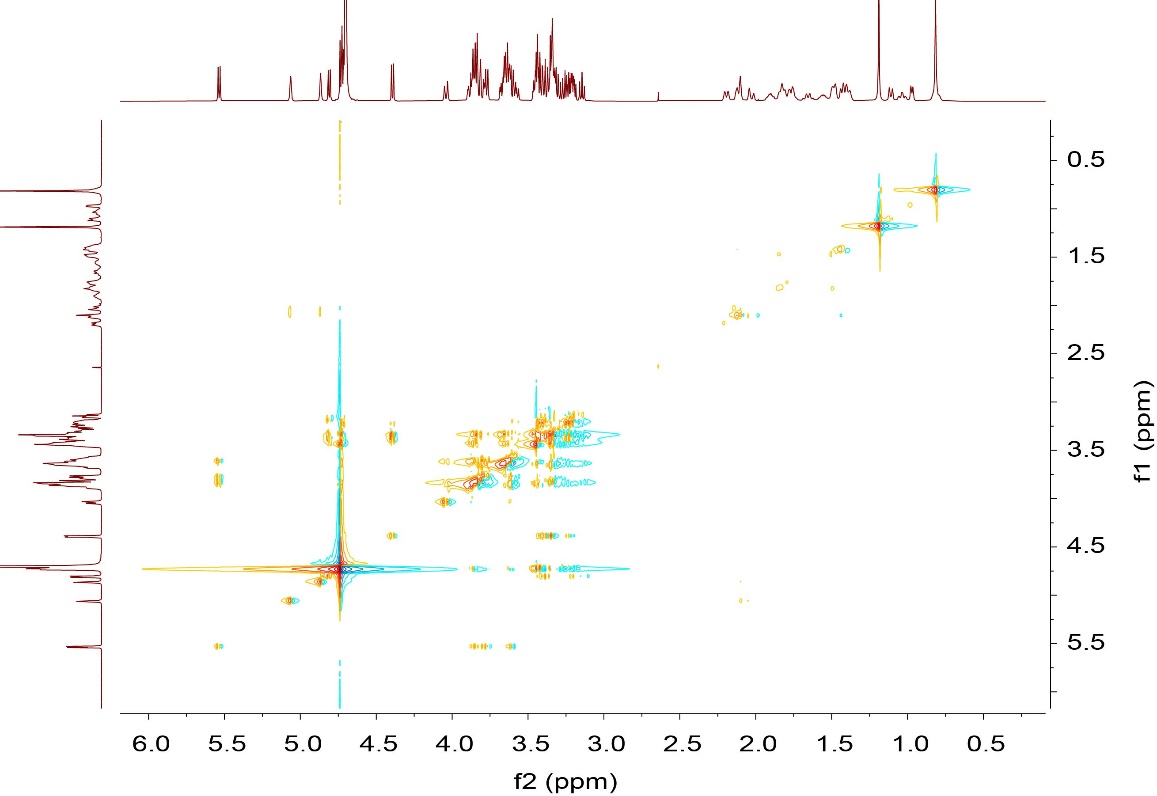


**Supplementary Figure 5.** ^1^H-^1^H TOCSY spectrum of Reb M2.


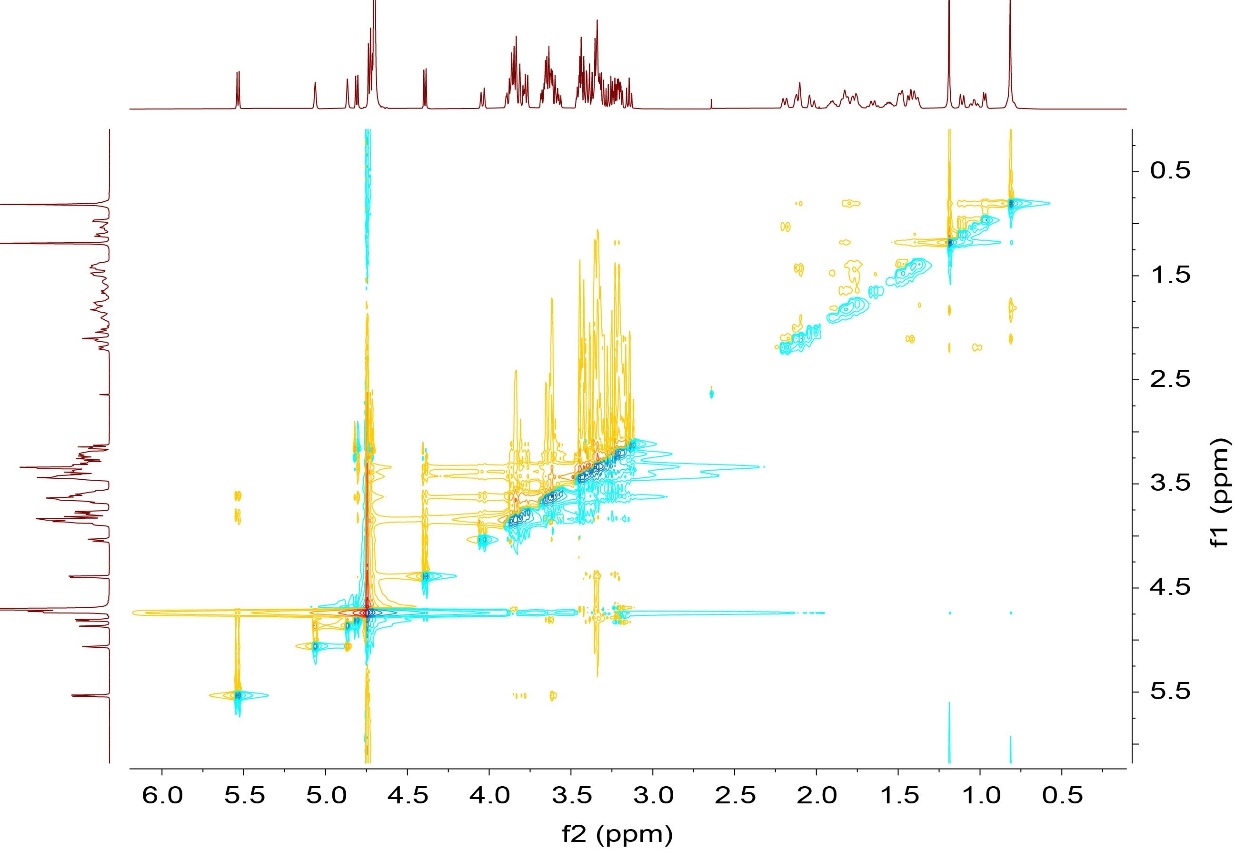


**Supplementary Figure 6.** ^1^H-^1^H ROESY spectrum of Reb M2.


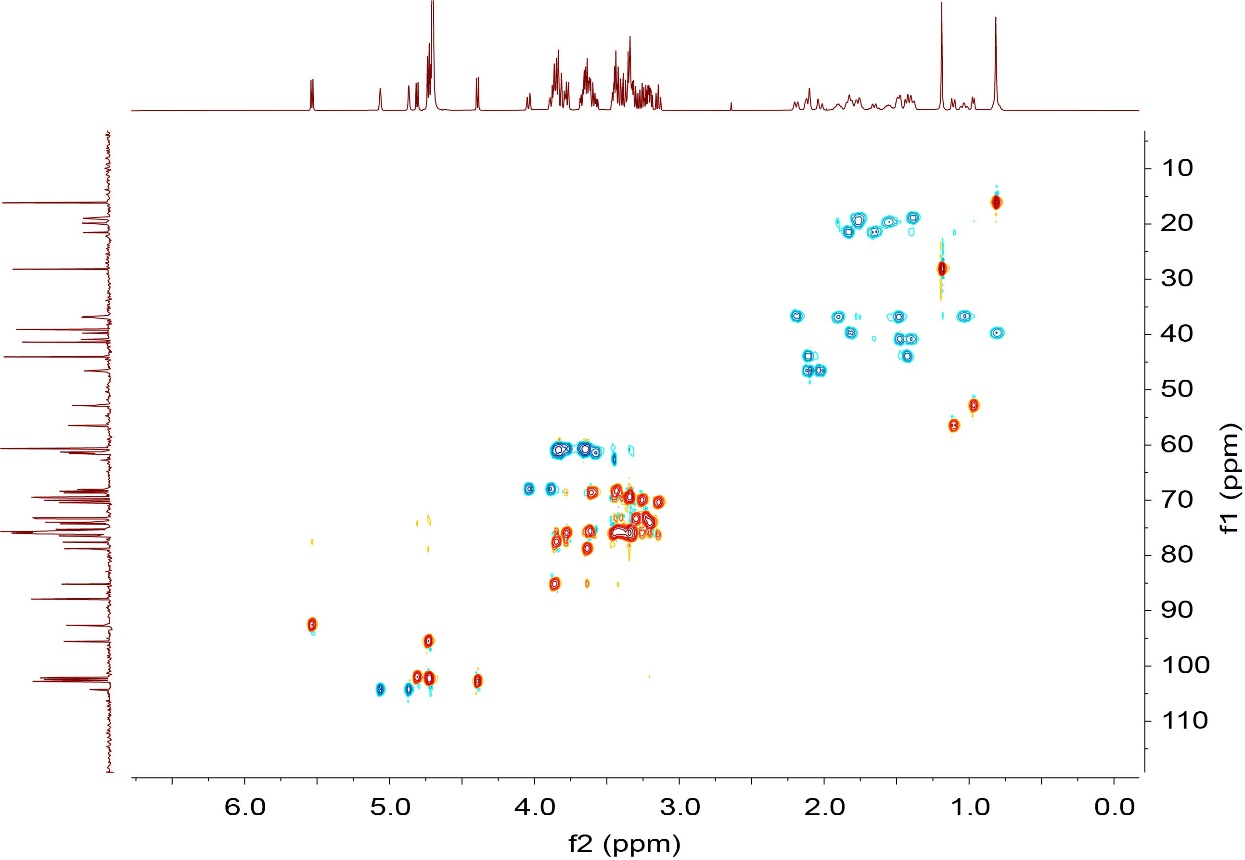


**Supplementary Figure 7.** ^1^H-^13^C HSQC spectrum of Reb M2.


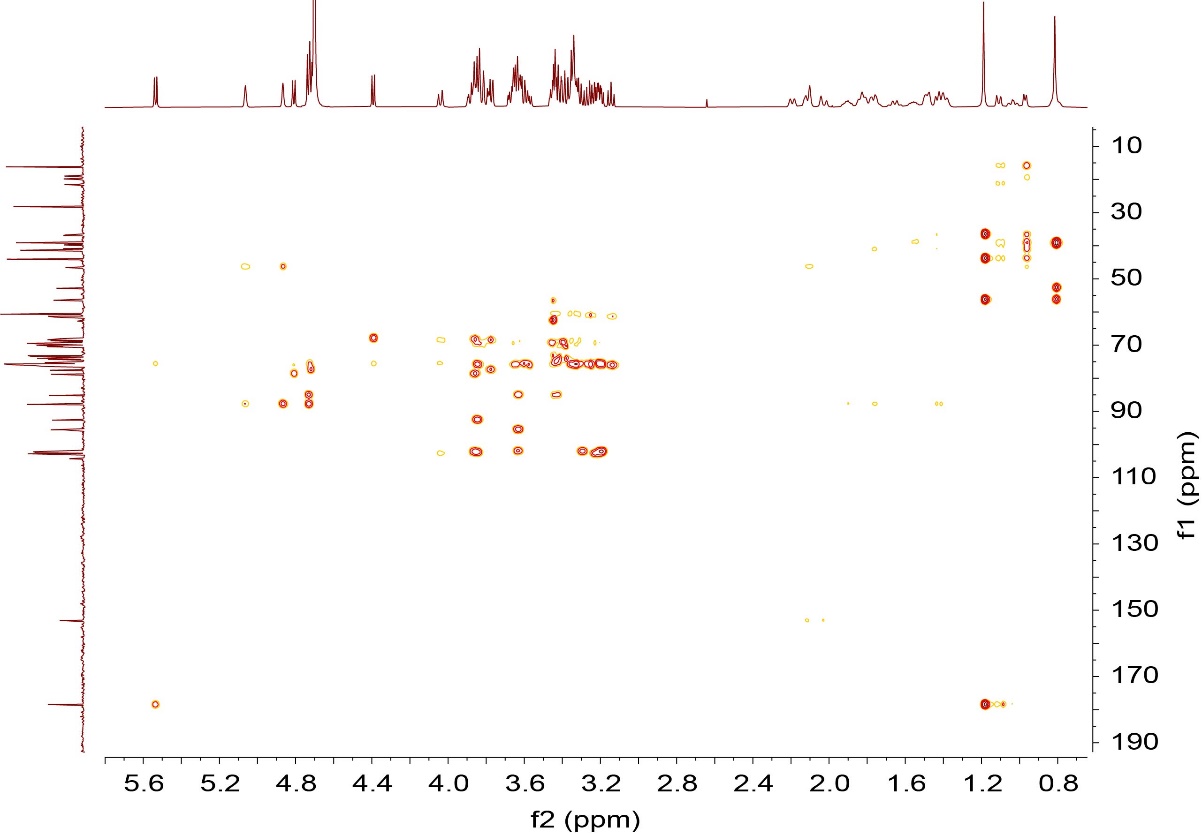


**Supplementary Figure 8.** ^1^H-^13^C HMBC spectrum of Reb M2.


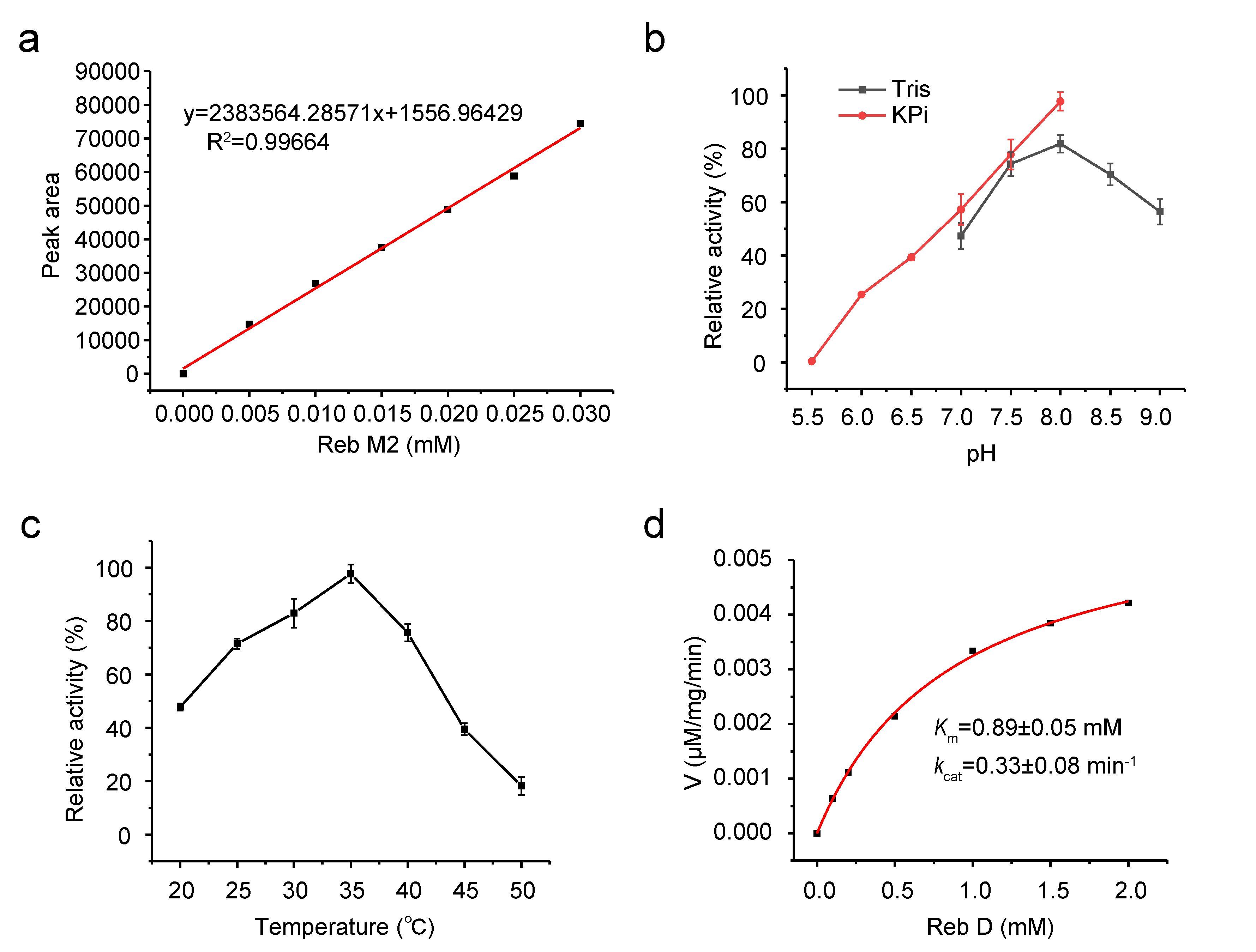


**Supplementary Figure 9.** Characterization of enzymatic properties of UGT94D1. (a) Standard curves of Reb M2. (b) Effect of pH on the activity of UGT94D1. (c) Effect of temperature on the activity of UGT94D1. (d) Kinetic parameters of UGT94D1.


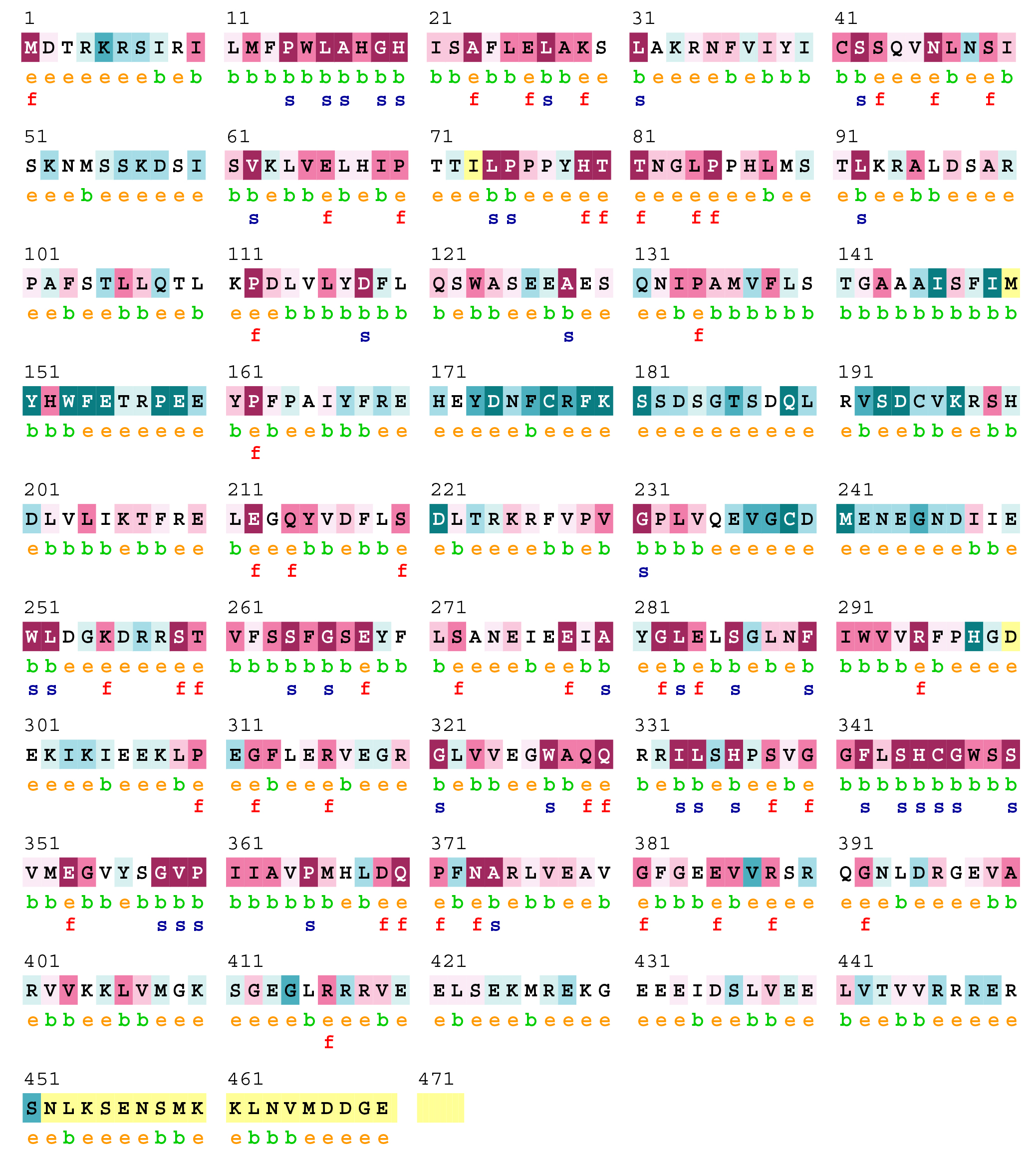


**Supplementary Figure 10.** Secondary structure prediction and sequence conservation analysis of UGT94D1.


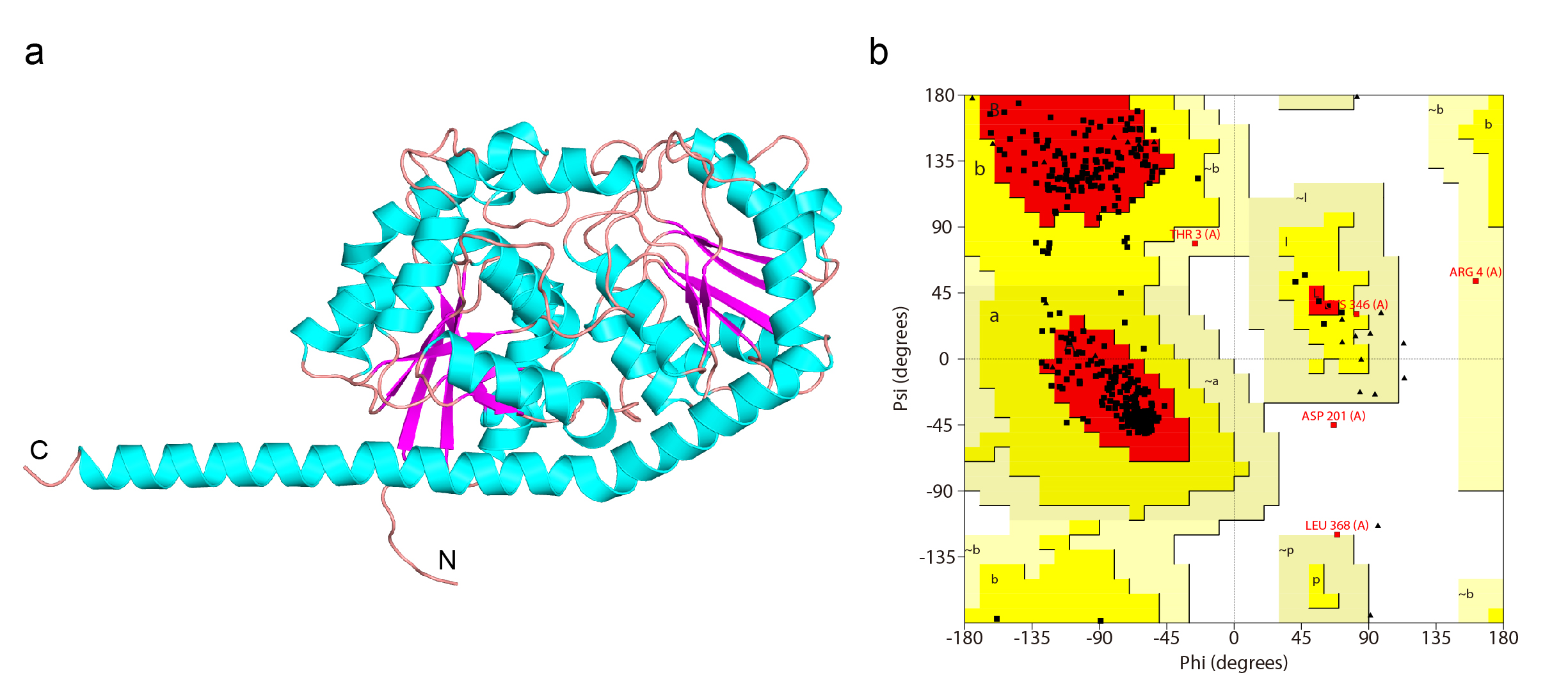


**Supplementary Figure 11.** Structural prediction and model validation of UGT94D1. (a) The structure of UGT94D1 predicted by Alphafold2. (b) Ramachandran plot analysis of predicted 3D structure of UGT94D1.


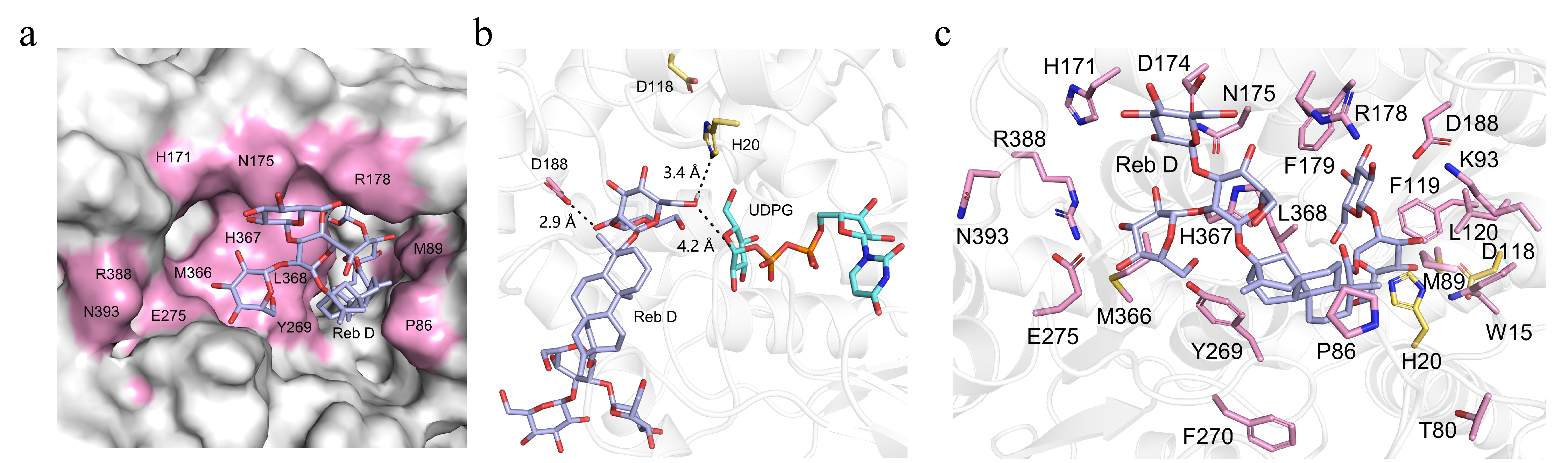


**Supplementary Figure 12**. Substrate-binding pocket analysis of UGT94D1. (a) Residues in UGT94D1 involved in hydrophobic interactions with Reb D based on molecular docking. (b) Residues in UGT94D1 responsible for the formation of hydrogen bond with Reb D. The black dash lines indicate the distances. The catalytic residues H20 and D118 were highlighted. (c) Residues in UGT94D1 (pink sticks) located around Reb D within 4 Å. Reb D and active sites (H20 and D118) are represented in purple and yellow sticks, respectively. The atoms oxygen and nitrogen are shown by the colors red and blue, respectively.

**Reference**

(1) Prakash, I.; Bunders, C.; Devkota, K. P.; Charan, R. D.; Ramirez, C.; Priedemann, C.; Markosyan, A. Isolation and characterization of a novel rebaudioside M isomer from a bioconversion reaction of rebaudioside A and NMR comparison studies of rebaudioside M isolated from Stevia rebaudiana Bertoni and Stevia rebaudiana Morita. *Biomolecules* **2014,** *4*, 374-89.
